# Supplementary material for: Influence of the extracellular domain size on the dynamic behavior of membrane proteins
Source: Biophys J. 2022 Sep 14;121(20):3826–36. doi: 10.1016/j.bpj.2022.09.010 (PMC9674980; doi:10.1016/j.bpj.2022.09.010)
Supplement: Document S1. Figures S1–S12 and Tables S1–S3 [file mmc1.pdf]

Biophysical Journal, Volume *121*

Supplemental information

Influence of the extracellular domain size on the dynamic behavior of membrane proteins

Cenk Onur Gurdap, Linda Wedemann, Taras Sych, and Erdinc Sezgin

Supplementary Data

Supplementary Figures

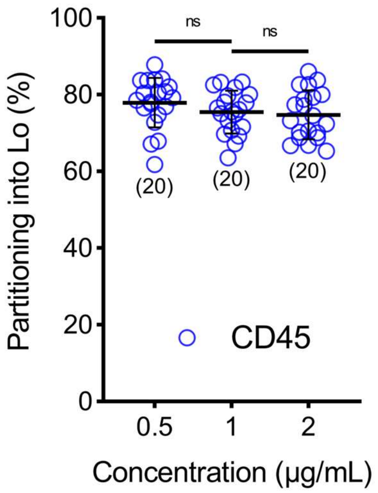

Supp. Fig. 1. The partitioning of CD45 (the largest protein) remains constant across varying protein concentrations. A non-parameterized ANOVA was used to determine significance (ns: non-significant). Horizontal lines show the mean and error bars represent the standard deviation. The number of data points obtained for each experiment are indicated on the graphs in parentheses.

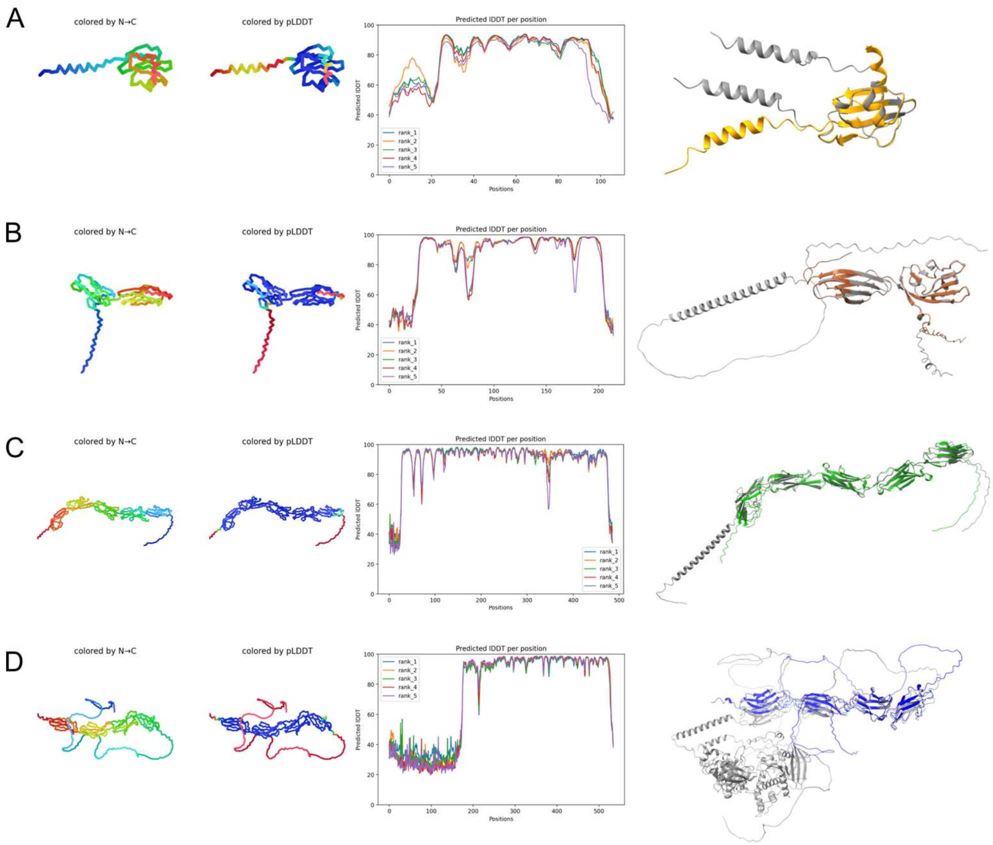

Supp. Fig. 2. ECD Structural models via ALPHAFOLD2. Model obtained via Collabfold, multiple sequence alignment performed via Jackhmmer, displayed is the highest ranked out of 5 models performed, with the LDDT graph for all models displayed next to the structures. Also shown are alignments to published structures for the whole protein from PDB, each being alphafold models, PDB code indicated with the protein as following: (A) CD59, AlphaFold: AF-P13987-F1, (B) CD2, AlphaFold: AF_P06729-F1, (C) ICAM-1, AlphaFold: AF-P05362-F1, (D) CD45, AlphaFold: AF-P08575-F1. Some of the structures cannot be resolved with Alphafold, presumably due to glycosylation.

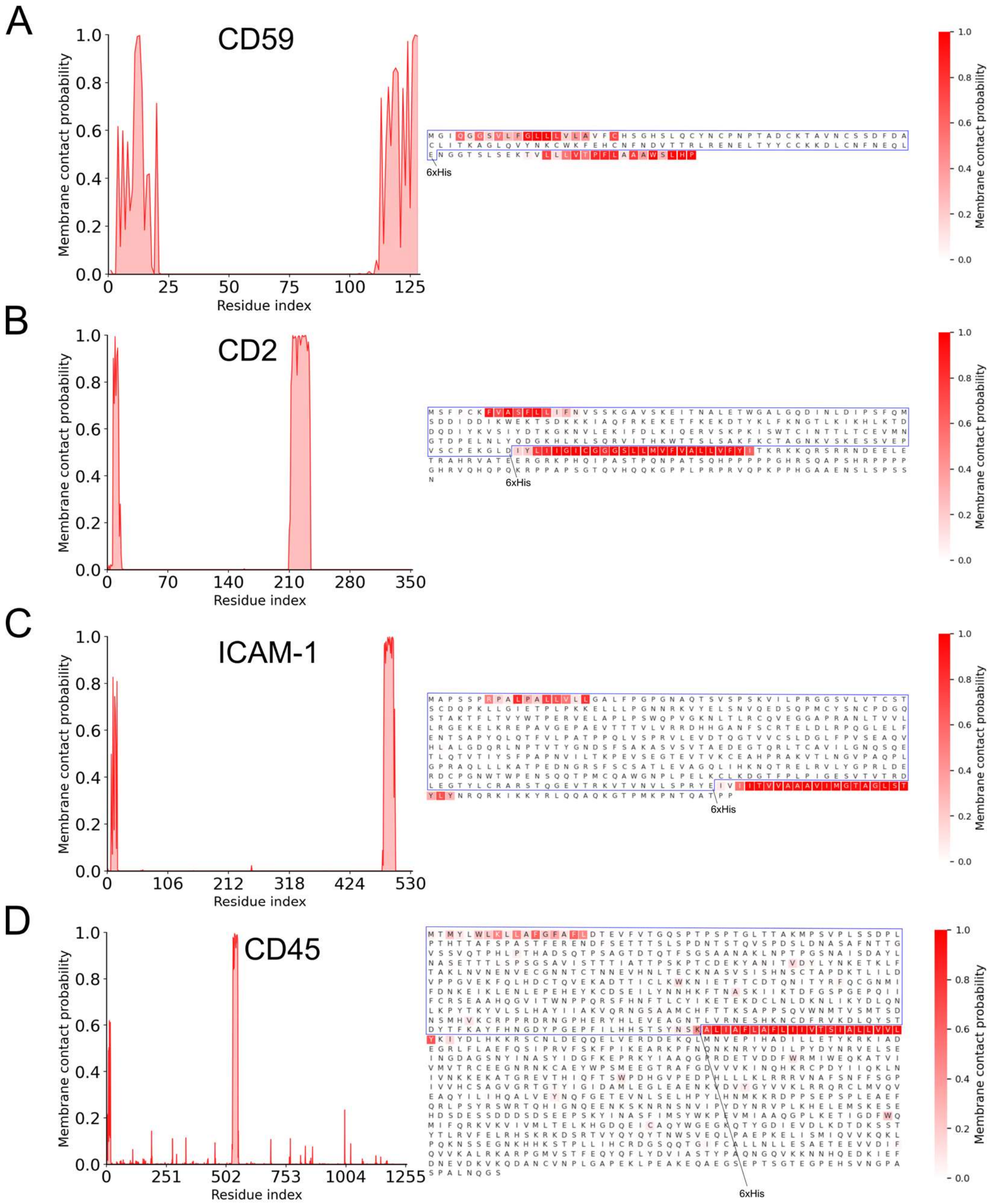

Supp. Fig. 3. Membrane contact probability. The analysis was done on full-length proteins and blue frames show extracellular domain of the proteins used in the study. 6xHis-tag in C-terminus is shown with arrow for each protein. Amino acids next the membrane anchoring sites can result in protein lipid interactions. Each protein has similar hydrophobic residues towards the far end of the proteins.

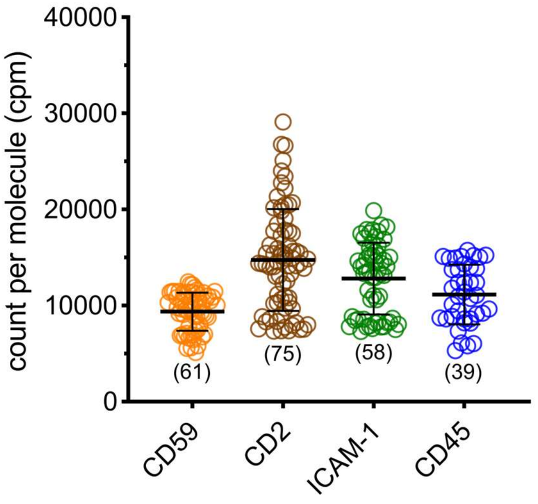

Supp. Fig. 4. Count per molecule for FCS measurement on GUVs. Horizontal lines show the mean and error bars represent the standard deviation. The number of data points obtained from experiments are indicated on the graphs in parentheses. Data does not show a trend that can correlate with diffusion or partitioning.

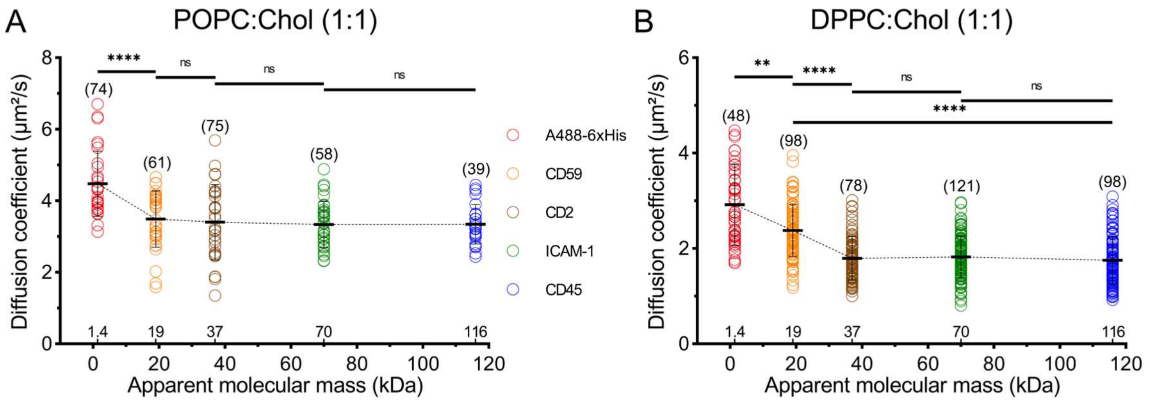

Supp. Fig. 5. Diffusion coefficients of the different ECDs in GUVs with (A) POPC:Chol (1:1) and (B) with DPPC/Chol (1:1). Student's t-test (two-tailed, non-parameterized) was used to determine significance (****P<0.0001; **P=0.001; ns: non-significant). Horizontal lines show the mean and error bars represent the standard deviation. The number of data points obtained from experiments are indicated on the graphs in parentheses.

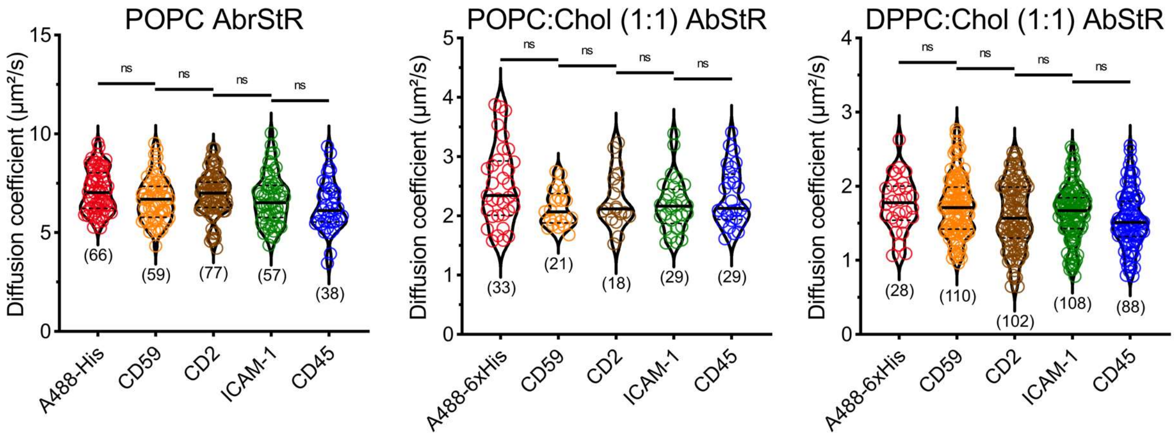

Supp. Fig. 6. Diffusion of membrane dye (AbStR-DPPE) in GUVs. Non-parameterized ANOVA was used to determine significance (ns: non-significant). Horizontal thick lines show the median and dotted lines represent the quartiles. The number of data points obtained from experiments are indicated on the graphs in parentheses.

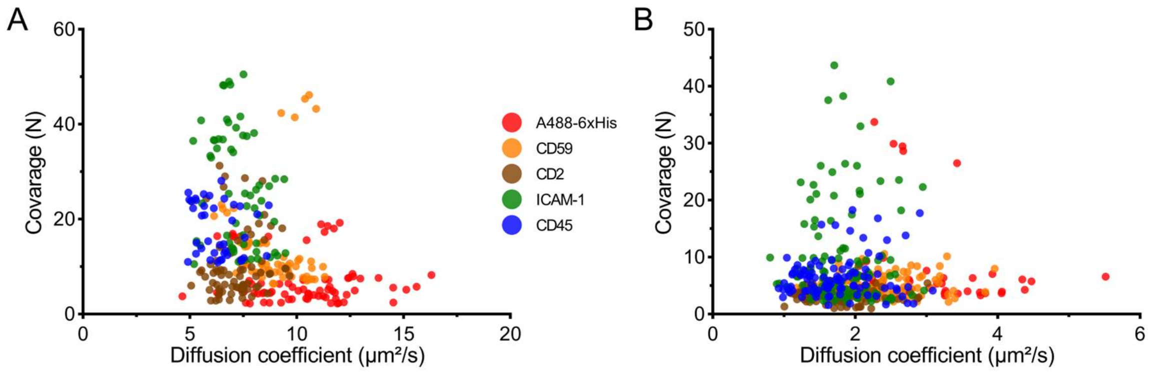

Supp Fig. 7. Number of molecules (coverage) vs diffusion time. Membrane coverage correlated to the diffusion coefficients obtained during the experiments for (A) POPC and (B) DPPC:Chol in GUVs. The plot shows no correlation between the mobility and membrane coverage, displaying that the experiments were performed under non-crowding affected conditions.

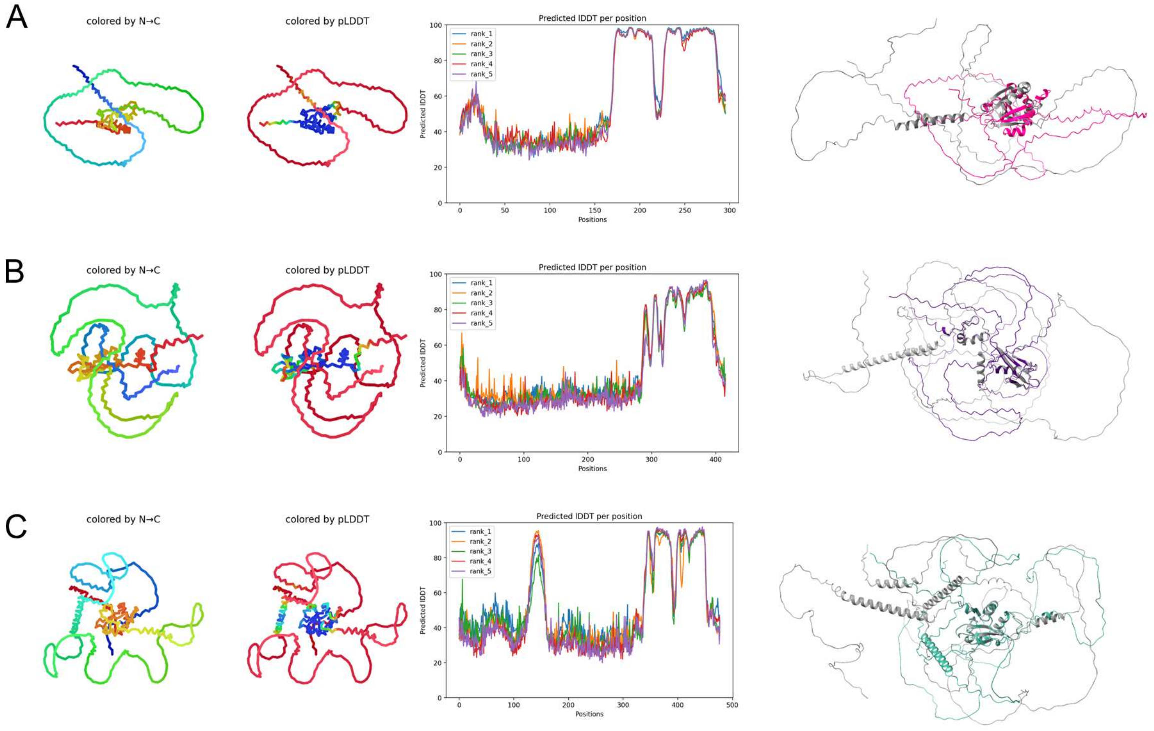

Supp. Fig. 8. ECD Structural models via ALPHAFOLD2. Model obtained via Collabfold, multiple sequence alignment performed via Jackhmmer, displayed is the highest ranked out of 5 models performed, with the LDDT graph for all models displayed next to the structures. Also shown are alignments to published structures for the whole protein's published Alphafold prediction. (A) CD34, AlphaFold: AF-P28906-F1, (A) CD34, AlphaFold: AF-P28906-F1, (B) PODXL, AlphaFold: AF-O00592-F1, (C) PODXL2, AlphaFold: AF-Q9NZ53-F1. Some of the structures cannot be resolved with Alphafold, presumably due to glycosylation.

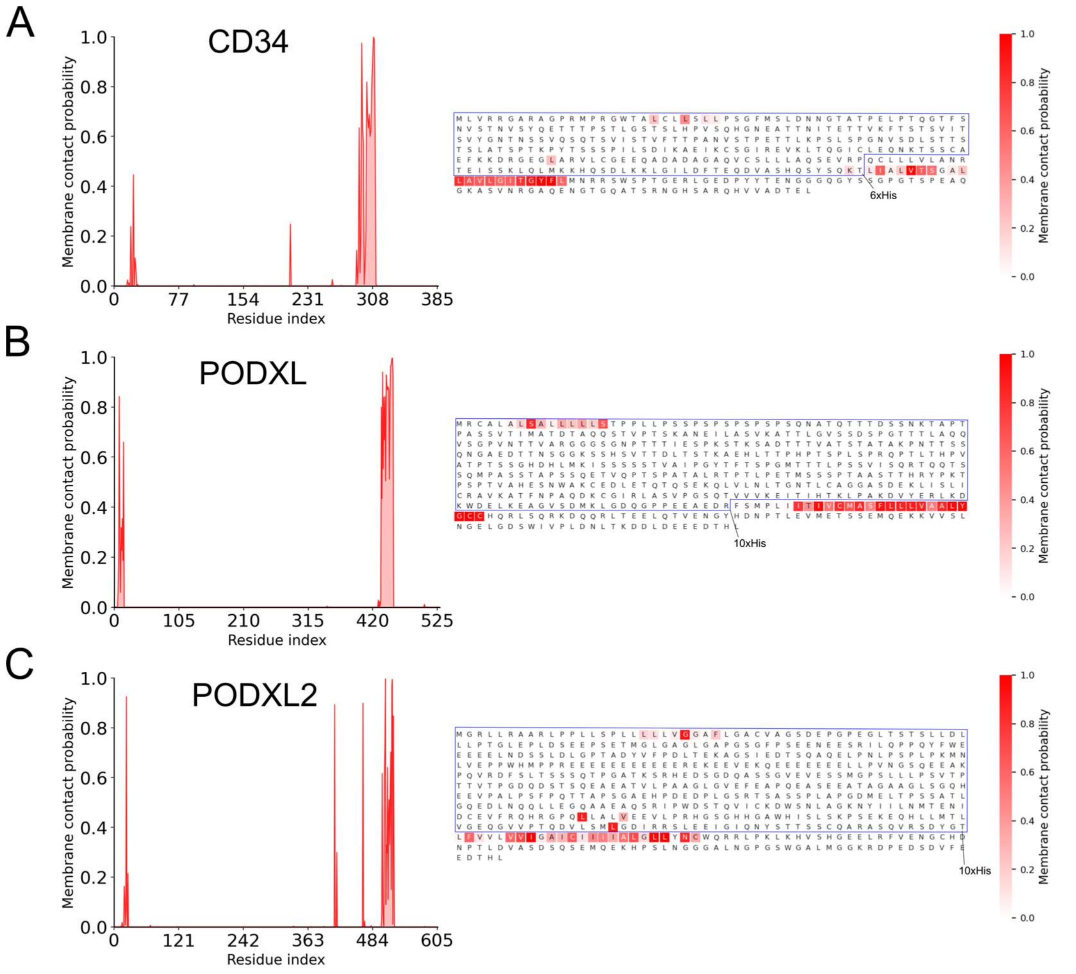

Supp. Fig. 9. Membrane contact probability. The analysis was done on the full-length proteins, and blue frames show extracellular domain of the proteins used in the study. The 6xHis-tag or 10xHis-tag at C-terminus is shown with arrow for each protein.

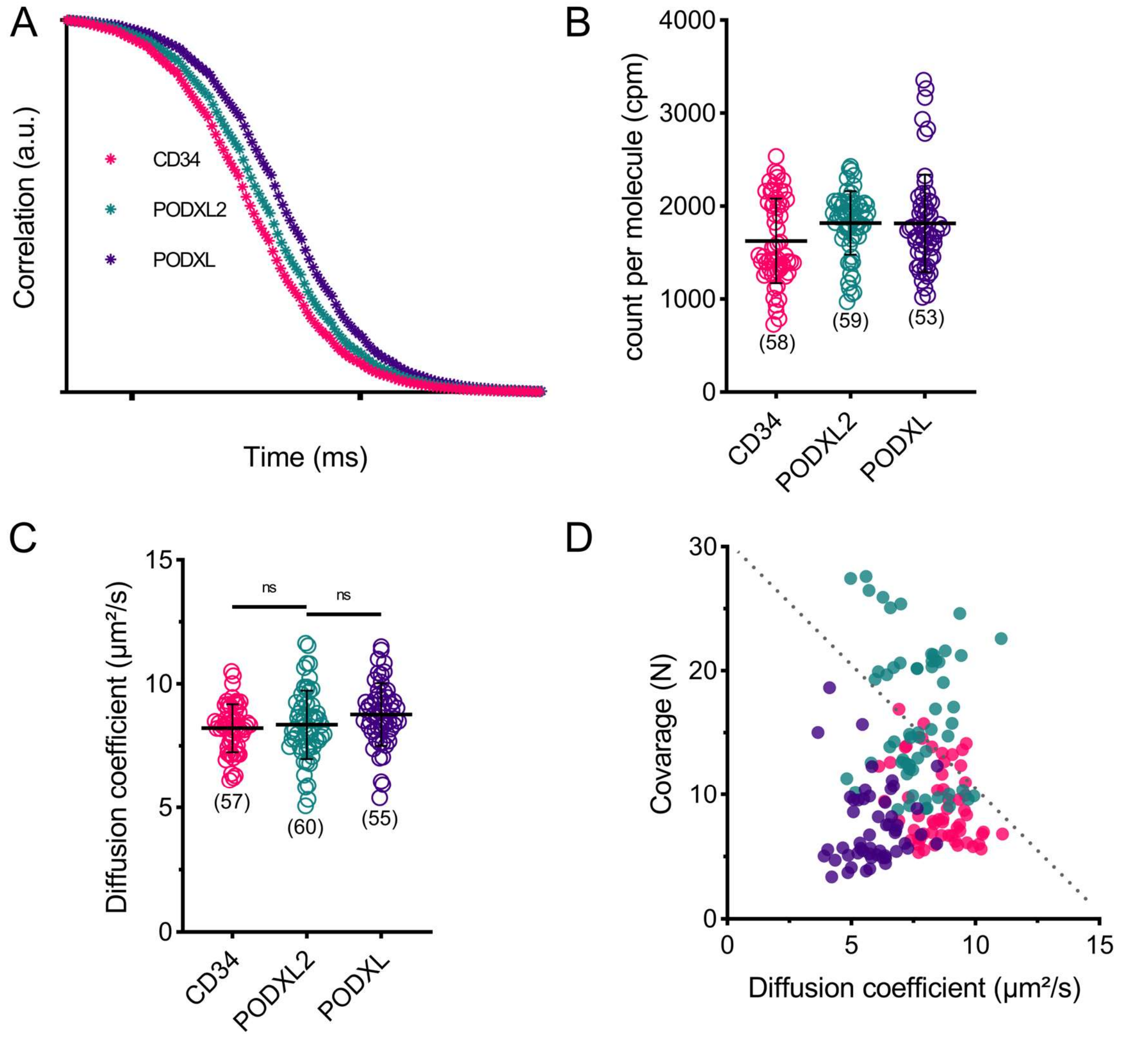

Supp. Fig. 10. Control experiments for CD34, PODXL2, and PODXL. (A) FCS curves. (B) Count per molecule for the FCS measurement in GUVs. (C) Diffusion of the membrane dye (AbStR-DPPE) in GUVs. (D) Number of molecules (coverage) vs diffusion time. Non-parameterized ANOVA was used to determine significance (ns: non-significant). Horizontal lines show the mean and error bars represent the standard deviation. The number of data points obtained from experiments are indicated on the graphs in parentheses.

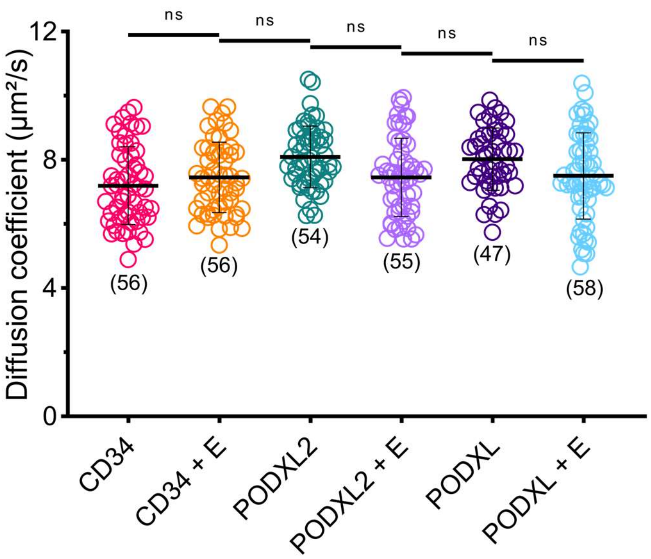

Supp. Fig. 11. Diffusion of membrane dye (AbStR-DPPE) in GUVs. Non-parameterized ANOVA was used to determine significance (ns: non-significant). Horizontal lines show the mean and error bars represent the standard deviation. The number of data points obtained from experiments are indicated on the graphs in parentheses. E stands for the enzyme treatment for the deglycosylation.

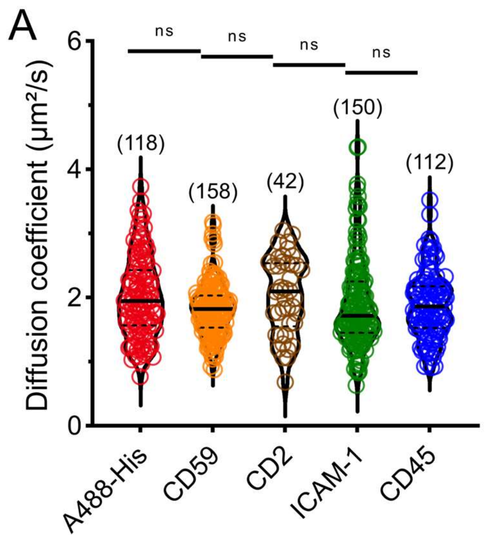

Supp. Fig. 12. Diffusion of membrane dye (AbStR-DPPE) in GPMVs. Non-parameterized ANOVA was used to determine significance (ns: non-significant). Horizontal thick lines show the median and dotted lines represent the quartiles. The number of data points obtained from experiments are indicated on the graphs in parentheses.

Supplementary Tables

Supp. Table 1: Molecular weight and required values for dye volume calculation for each of the ECDs investigated.

	Amino acids	MW_p (kDa)	ME	C_d (mg/mL)	V_d (µL)	Extinction coefficient ($M^{-1}cm^{-1}$)	C_p (mg/mL)
A488-6xHis	6	1.4	-	-	-	-	0.0025
CD59	101	19	3	5	1	12085	0.36
CD2	209	37	3	0.5	4	28210	0.32
ICAM-1	480	70	5	0.5	3	41785	0.43
CD45	529	116	5	0.5	3	51310	0.43
CD34	270	50	8	1	8.9	11835	~1
PODXL	405	165	8	1	6	15720	~1
PODXL2	468	110	8	1	5	35200	~0.8

Supp. Table 2: Alphafold2 (colabfold) parameters chosen during the model generation.

Parameter	Option chosen
**Search against genetic database**	
Msa_method	Jackhammer
Add_custom_msa	no
Pair_mode	Unpaired
Pair_cov	50
Pair-qid	20
**Filtering options**	
trim	no
Cov	no
Qid	no
**Run alphafold**	
Rank_by	pLDDT
Use turbo	Yes
Max_msa	512:1024
Num_models	5
Use_ptm	Yes
Num_ensemble	1
Max_recycles	3
Tol	o
Is_training	No
Num_samples	1
Subsample_msa	Yes
**Refine structures with Amber relax**	
Num_relax	None

Supp. Table 3: Amino acid sequences of the ECDs used in the project. Grey colors show the N-terminal 6xHis-Tag or 10xHis-tag.

ECD	Sequence
CD59	MGIQGGSVLFGLLLVLAVFCHSGHSLQCYNCPNPTADCKTAVNCSSDFDACLITKAGLQV<br>YNKCWKFEHCNFNDVTTRLRENELTYYCCKKDLCNFNEQLE HHHHHH
CD2	MSFPCKFVASFLLIFNVSSKGAVSKEITNALETWGALGQDINLDIPSFQMSDDIDDIKWE<br>KTSDKKKIAQFRKEKETFKEKDTYKLFKNGTLKIKHLKTDDQDIYKVSIYDTKGKNVLEK<br>IFDLKIQERVSKPKISWTCINTTLTCEVMNGTDPELNLYQDGKHLKLSQRVITHKWTTSL<br>SAKFKCTAGNKVSKESSVEPVSCPEKGLHHHHHH
Icam-1	MAPSSPRPALPALLVLLGALFPGPGNAQTSVSPSKVILPRGGSVLVTCSTSCDQPKLLGI<br>ETPLPKKELLLPGNNRKVYELSNVQEDSQPMCYSNCPDGQSTAKTFLTVYWTPERVELAP<br>LPSWQPVGKNLTLRCQVEGGAPRANLTVVLLRGEKELKREPAVGEPAEVTTTVLVRRDHH<br>GANFSCRTELDLRPQGLELFENTSAPYQLQTFVLPATPPQLVSPRVLEVD TQGTVVCSLD<br>GLFPVSEAQVHLALGDQRLNPTVTYGNDSFSAKASVSVTAEDEGTQRLTCAVILGNQSQE<br>TLQTVTIYSFPAPNVILTKPEVSEGTEVTVKCEAHPRAKVTLNGVPAQPLGPRAQLLLKA<br>TPEDNGRSFSCSATLEVAGQLIHKNQTRELRVLYGPRLDERDCPGNWTWPENSQQTPMCQ<br>AWGNPLPELKCLKDGTFPLPIGESVTVTRDLEGTYLCRARSTQGEVTRKVTVNVLSPRY H<br>HHHHH
CD45	MTMYLWLKLLAFGFAFLDTEVFVTGQSPTPSPTGLTTAKMPSVPLSSDPLPTHTTAFSPA<br>STFERENDFSETTTSLSPDNTSTQVSPDSLDNASAFNTTGVSSVQTPHLPTHADSQTPSA<br>GTDTQTFSGSAANAKLNPTPGSNAISDAYLNASETTTLSPSGSAVISTTTIATTPSKPTC<br>DEKYANITVDYLYNKETKLFTAKLNVNENVECGNNTCTNNEVHNLTECKNASVSISHNSC<br>TAPDKTLILDVPPGVEKFQLHDCTQVEKADTTICLKWKNIETFTCDTQNITYRFQCGNMI<br>FDNKEIKLENLEPEHEYKCDSEILYNNHKFTNASKIIKTDFGSPGEPQIIFCRSEAAHQG<br>VITWNPPQRSFHNFTLCYIKETEKDCLNLDKNLIKYDLQNLKPYTKYVLSLHAYIIAKVQ<br>RNGSAAMCHFTTKSAPPSQVWNMTVSMTSDNSMHVKCRPPRDRNGPHERYHLEVEAGNTL<br>VRNESHKNCDFRVKDLQYSTDYTFKAYFHNGDYPGEPFILHHSTSYNSK HHHHHH
CD34 prec.	MLVRRGARAGPRMPRGWTALCLLSLLPSGFMSLDNNGTATPELPTQGTFSNVSTNVSYQE<br>TTTPSTLGSTSLHPVSQHGNEATTNITETTVKFTSTSVITSVYGNTNSSVQSQTSVISTV<br>FTTPANVSTPETTLKPSLSPGNVSDLSTTSTSLATSPTKPYTSSSPILSDIKAEIKCSGI<br>REVKLTQGICLEQNKTSSCAEFKKDRGEGLARVLCGEEQADADAGAQVCSLLLAQSEVRP<br>QCLLLVLANRTEISSKLQLMKKHQSDLKKLGILDFTEQDVASHQSYSQKT HHHHHH
PODXL	SPSPSPSPSPSQNATQTTTDSSNKTAPTPASSVTIMAT DTAQQSTVPTSKANEILASVKA<br>TTLGVSSDSPGTTTLAQQVSGPVNTTVARGGGSGNPTT TIESPKSTKSADTTTVATSTAT<br>AKPNTTSSQNGAEDTTNSGGKSSHSVTTDLTSTKAEHL TTPHPTSPLSPRQPTLTHPVAT<br>PTSSGHDHLMKISSSSSTVAIPGYTFTSPGMTTTLPSS VISQRTQQTSSQMPASSTAPSS<br>QETVQPTSPATALRTPTLPETMSSSPTAASTTHRYPKT PSPTVAHESNWAKCEDLETQTQ<br>SEKQLVLNLTGNTLCAGGASDEKLISLICRAVKATFNP AQDKCGIRLASVPGSQTVVVKE<br>ITIHTKLPAKDVYERLKDKWDELKEAGVSDMKLGDQGP PEEAEDRHHHHHHHHHH
PODXL2	GSDEPGPEGLTSTSLLDLLLPTGLEPLD SEEPSETMGLGAGLGAPGSGFPSEENEESRIL<br>QPPQYFWEEEEELNDSSLDLGPTADYVF PDLTEKAGSIEDTSQAQELPNLPSPLPKMNLV<br>EPPWHMPPREEEEEEEEEEEREKEEVEK QEEEEEEELLPVNGSQEEAKPQVRDFSLTSSS<br>QTPGATKSRHEDSGDQASSGVEVESSMG PSLLLPSVTPTTVTPGDQDSTSQEAEATVLPA<br>AGLGVEFEAPQEASEEATAGAAGLSGQH EEVPALPSFPQTTAPSGAEHPDEDPLGSRTSA<br>SSPLAPGDMELTPSSATLGQEDLNQQLL EGQAAEAQSRIPWDSTQV ICKDWSNLAGKNYI<br>ILNMTENIDCEVFRQHRGPQLLALVEEV LPRHGSGHHGAWHISLSKPSEKEQHLLMTLVG<br>EQGVVPTQDVLSMLGDIRRSLEEIGIQN YSTTSSCQARASQVRSDYGT HHHHHHHHHH
