# Supplementary material for: Influence of the extracellular domain size on the dynamic behavior of membrane proteins
Source: Biophys J. 2022 Sep 14;121(20):3826–36. doi: 10.1016/j.bpj.2022.09.010 (PMC9674980; doi:10.1016/j.bpj.2022.09.010)
Supplement: Document S2. Article plus supporting material [file mmc2.pdf]

Biophysical Journal
Article

Biophysical Society

Influence of the extracellular domain size on the dynamic behavior of membrane proteins

Cenk Onur Gurdap,[1] Linda Wedemann,[1] Taras Sych,[1] and Erdinc Sezgin[1,*]
[1]Science for Life Laboratory, Department of Women's and Children's Health, Karolinska Institutet, Solna, Sweden

ABSTRACT The dynamic behavior of plasma membrane proteins mediates various cellular processes such as cellular motility, communication, and signaling. It is widely accepted that the dynamics of the membrane proteins is determined either by the interactions of the transmembrane domain with the surrounding lipids or by the interactions of the intracellular domain with cytosolic components such as cortical actin. Although initiation of different cellular signaling events at the plasma membrane has been attributed to the extracellular domain (ECD) properties recently, the impact of ECDs on the dynamic behavior of membrane proteins is rather unexplored. Here, we investigate how ECD properties influence protein dynamics in the lipid bilayer by reconstituting ECDs of different sizes or glycosylation in model membrane systems and analyzing ECD-driven protein sorting in lipid domains as well as protein mobility. Our data show that increasing the ECD mass or glycosylation leads to a decrease in ordered domain partitioning and diffusivity. Our data reconcile different mechanisms proposed for the initiation of cellular signaling by linking the ECD size of membrane proteins with their localization and diffusion dynamics in the plasma membrane.

SIGNIFICANCE We studied how the size and glycosylation of the proteins influences their dynamic behavior in a lipid bilayer by reconstituting the extracellular domains (ECDs) of different sizes or glycosylation in model membrane systems and analyzing their sorting into lipid domains as well as their mobility. We observe that increasing the ECD apparent mass leads to a decrease in membrane-ordered domain partitioning and diffusivity. Our data reconcile multiple mechanisms proposed for the initiation of cellular signaling by linking the ECD properties of membrane proteins with their localization and diffusion dynamics in the plasma membrane.

INTRODUCTION

The plasma membrane (PM) is a complex and dynamically heterogeneous system consisting of a lipid bilayer populated with various lipids and membrane proteins (MPs). About 40% of the human proteome is membrane-associated proteins (1) that are either integrally embedded into the PM or peripherally associated by interactions with the headgroups of lipids. Integral MPs are embedded in the membrane via one or multiple transmembrane domain(s) (TMD), with extramembraneous hydrophilic domains extending into the cytosol (intracellular domain [ICD]) or into the extracellular environment (extracellular domain [ECD]).

Cellular signaling events are often triggered at the PM via ligand-receptor interactions. For example, immune cell receptors are spatially reorganized and phosphorylated when interacting with their target ligands, eventually initiating a downstream signaling cascade. Multiple mechanisms are proposed to explain the initial protein reorganization that leads to the activation (phosphorylation) of the receptors. Partitioning of signaling proteins into membrane domains (2), their ECD size (3–5), their allosteric interactions with lipids (6), and their mobility altered by the ligands (7,8) are some examples of these mechanisms. Although there is compelling evidence for each of these mechanisms for immune signaling, the dominating mechanism is still unclear. In this work, we investigated whether these seemingly unrelated mechanisms can be, in fact, intertwined. To this end, by using model membranes and fluorescence correlation spectroscopy (FCS), we investigated the relationship between the ECD size or glycosylation of various immune signaling proteins, their molecular diffusion, and their domain partitioning.

Model membranes are widely used as a synthetic proxy to investigate biophysical aspects of the PM, such as membrane domain formation (9). In these simple model membrane

Submitted January 18, 2022, and accepted for publication September 12, 2022.

*Correspondence: erdinc.sezgin@ki.se

Cenk Onur Gurdap and Linda Wedemann contributed equally to this work.

Editor: Rumiana Dimova.

https://doi.org/10.1016/j.bpj.2022.09.010

© 2022 Biophysical Society.

This is an open access article under the CC BY license (http://creativecommons.org/licenses/by/4.0/).

systems, saturated lipids form a liquid-ordered (L_o) phase with the help of cholesterol, and unsaturated lipids form a liquid-disordered (L_d) phase. Different artificial or cell-derived model systems are available to study the phase separation phenomenon (9). Giant unilamellar vesicles (GUVs) and giant PM vesicles (GPMVs) are commonly used cell-sized spherical free-standing lipid bilayers. While GPMVs are cell derived and thereby inherently more complex in lipid and protein compositions, GUVs are artificial systems with finely controlled composition. Both systems could display the coexisting phases to mimic the lateral heterogeneity of the PM (10).

Proteins were shown to partition into one of these lipid phases preferentially. Such selective protein partitioning can induce lipid environment-dependent conformational change and promote lipid-driven protein associations, crucial for protein function, such as immune cell function and viral dynamics (11–14). Recent studies showed that TMD length, lipid-accessible surface area of TMD, and the addition of fatty acid molecules such as palmitoylation at the TMD can influence protein partitioning in membranes (15–17). Glycosylphosphatidylinositol (GPI)-anchored proteins partition based on the structure of their lipid anchors (18). Critical factors in phase separation and protein partitioning are reviewed in detail in (19,20). While there is extensive research on the influence of TMD properties on phase partitioning of proteins (17), the role of ECD properties (e.g., length, molecular weight, and glycosylation) on partitioning is largely unexplored. Some of the earlier work performed using biochemical methods suggested a nonexisting role of ECDs in membrane domain partitioning of proteins, such as CD4 (21). In recent years, however, the role of ECDs on the membrane structure and dynamics have been addressed using state-of-the-art methods. For example, it was shown that protein assemblies at the extracellular side of the membrane induce membrane reorganization, such as membrane bending (22), tube formation (23), concomitant polymerization (24), or domain dissolution (25). Moreover, ECD size has been one of the major players in immune synapse formation (3), continuously being studied in the context of lipid remodeling (26). Finally, different lengths of DNA oligonucleotides showed different phase partitioning in phase-separated synthetic vesicles (27). Altogether, this evidence necessitates a thorough study on the effect of ECDs on the partitioning behavior of the MPs.

To exhibit their function, MPs need to be dynamic, since their lateral mobility allows them to interact with other proteins and thereby form complexes. Therefore, measuring the lateral mobility of proteins is crucial to gain a mechanistic understanding of their function. Influenced by the compositional complexity, the dynamics of MPs are not only governed by simple Brownian motion, but it is rather more complex, mediated by lipid domains, protein-protein interactions, and cytoskeletal elements (28–34). According to the established Saffman and Delbrück model, when a molecule is anchored to the membrane, its diffusion depends on membrane viscosity (η_m), membrane thickness (h), and surrounding bulk fluid viscosity (η_f) and weakly on the radius of the TMD (R) (k_B is the Boltzman constant, γ is the Euler's constant, and T is temperature) (35):

$$D_{S\&D} = \frac{k_B T}{4\pi h \eta_m}\left(\ln\left(\frac{h\eta_m}{R\eta_f}\right) - \gamma\right). \tag{1}$$

There have been several corrections to this model (36–43). However, these corrections are mainly focused on the impact of lipid environment, TMD, and ICD of the proteins on lateral diffusion, with the exception of GPI-anchored proteins, where some hints of the influence of extramembraneous size on diffusion were observed (44,45). Recently, Houser et al. suggested a correlation between the ECD molecular weight and diffusion under crowded conditions, showing that larger proteins display a slower diffusion due to larger area coverage (crowding) (46). Furthermore, computational simulations (47) and experimental methods (48) have predicted that the diffusion of MPs might also be influenced by their ECDs. Therefore, it is crucial to reveal how ECD properties influence the diffusion of MPs and to develop new models taking this role into consideration.

In this work, we reconstituted polyhistidine-tagged ECDs of different sizes or glycosylation in model membranes functionalized with nickel-chelating lipids. In this setting, TMDs and ICDs are absent, hence the impact of ECD properties on protein behavior can directly be elucidated. To monitor the phase partitioning of ECDs, we used phase-separated GUVs, and interestingly, we found that protein sorting to the L_o phase decreases with increasing ECD mass. Furthermore, we studied ECD mobility in GUVs of different compositions and GPMVs. We found that protein mobility also decreases as the mass of the ECD increases. These changes correlate perfectly with the apparent mass of the proteins rather than their predicted mass by amino acid sequence. Finally, our data showed that glycosylation is a critical factor that influences both the partitioning and diffusion of proteins. When glycosylation was removed from proteins, they exhibited higher L_o partitioning and faster diffusion. Overall, our data suggest a critical role of the ECD properties on MP behavior in the PM and pave the way to a complete understanding of MP dynamics that also takes interaction with the extracellular matrix and glycocalyx into account.

MATERIALS AND METHODS

Lipids, proteins, and fluorescent probes

1-Palmitoyl-oleoyl-sn-glycero-phosphocholine (POPC), 1,2-Dipalmitoyl-sn-glycero-3 phosphocholine (DPPC), 1,2-Dioleoyl-sn-glycero-3-phosphocholine (DOPC), sphingomyelin (SM), cholesterol (Chol), 1,2-Dioleoyl-sn-glycero-3-[(N-(5-amino-1-carboxypentyl)iminodiacetic acid)succinyl] (nickel salt) (18:1 DGS-NTA(Ni)), and 1,2-Dipalmitoyl-sn-glycero-3-[(N-(5-amino-1-carboxypentyl)iminodiacetic acid)succinyl] (nickel salt) were

obtained from Avanti Polar Lipids (Alabaster, AL, USA). Lipid stocks were stored in chloroform under nitrogen at −20°C.

ECD fragments (modified C terminally with 6xHis tag) of cluster of differentiation (CD) 59, (cat. no. 12474-H08H), CD2 (cat. no. 10982-H08H), intercellular adhesion molecule (ICAM-1) (cat. no. 10346-H080H), CD45 (cat. no. 16884-H08H), and CD34 (cat. no. 10103-H08H) were purchased from Sino Biological (Beijing, China). ECD fragments (modified C terminally with 10xHis tag) podocalyxin (PODXL) (cat. no. 1658-PD) and PODXL2 (cat. no. 1524-EG) were obtained from R&D Systems (Minneapolis, MN, USA). The ECD fragments were dissolved in ultrapure water to a concentration of 0.5 mg/mL and aliquoted in 50 μL, snap frozen, and stored at −80°C. For immediate usage, protein solutions were stored at 4°C avoiding freeze/thaw cycles. 6xHis-tagged Alexa 488 (A488-6xHis), 1.2 μg/mL, was custom made by Cambridge Research Biochemicals (Billingham, UK). Abberior STAR Red-1,2-Dipalmitoyl-sn-glycero-3-phosphatidylethanolamine (AbStR-DPPE) was obtained from Abberior GmbH (Göttingen, Germany).

For protein labeling, Alexa Fluor 488 NHS Ester (A488) was purchased from Thermo Fisher Scientific (Waltham, MA, USA) and performed according to NHS labeling protocol. Briefly, dye solution in DMSO (concentration not lower than 10 mg/mL) was added to protein solution in pure water (0.5 mg/mL; 1 mg/mL for CD34, PODXL, and PODXL2) and incubated for 1 h at 300 Rpm in the presence of 100 mM $NaHCO_3$ (pH 8.3). Molar excess of dyes for each protein is displayed in Table S1. Purification was performed using spin columns (7 kDa exclusion limit, 0.5 mL Zeba spin desalting columns, Thermo Fisher Scientific). After purification, the protein concentration (C_p final) was measured via nanodrop with the knowledge of the individual extinction coefficients, estimated from the Uniprot amino-acid sequences using the ProtParam tool by Expasy (Table S1).

For deglycosylation treatment of the proteins, His-tagged CD34, PODXL, and PODXL2 were mixed with Protein Deglycosylation Mix II (New England Biolabs, Ipswich, MA, USA, #P6044S). The suggested protocol by the supplier was used with the exception of incubation time (the proteins were only incubated at room temperature [RT] for 20 h).

Preparation of model membranes

GUVs were produced via the electroformation method. A lipid mixture was dissolved in chloroform-methanol (1 mg/mL POPC, POPC:Chol [1:1], DPPC:Chol [1:1], or DOPC:SM:Chol [2:2:1, phase separated]), all with 1 mol % of Ni-NTA, and was spread on platinum wires and left for the solvent to evaporate. It was then dipped into 300 mM sucrose solution in a custom-build, Teflon-coated electroformation chamber. Electroformation was performed for 1 h with 10 Hz AC field (2V) followed by 30 min in 2 Hz (DG822, Rigol) for vesicle release from the wires. For the DPPC and DOPC:SM:Chol mixtures, an external heat source was used at 55°C for the electroformation, and the produced GUVs were then slowly cooled down to RT.

GPMVs were isolated from Chinese hamster ovary (CHO) cells, which were grown to about 70% confluency in Dulbecco's modified Eagle medium media supplemented with 10% fetal bovine serum (Sigma-Aldrich, Burlignton, MA, USA) and 1% L-glutamine or from HeLa cells with about 70% confluency (in Dulbecco's modified Eagle medium supplemented with 10% fetal bovine serum and 1% L-glutamine). Cells were prepared 2 days before the experiments by seeding them onto 6-well cell culture plates. They were then washed twice with 2 mL GPMV buffer (10 mM HEPES, 2 mM CaCl2, and 150 mM NaCl [pH 7.4]) and incubated in 1 mL GPMV buffer for 2 h. To form vesicles from CHO cells, the buffer was additionally supplemented with 25 mM paraformaldehyde (Sigma-Aldrich) and 2 mM 1,4-dithiothreitol (DTT; Sigma-Aldrich). To form phase-separated vesicles, HeLa cells were used to isolate GPMVs, where GPMV buffer was supplemented with 25 mM paraformaldehyde and 25 mM DTT.

To insert Ni-NTA lipids into GPMVs, the supernatant containing GPMVs was extracted. In an Eppendorf tube, the chloroform from 20 μg Ni-NTA lipid (5 mg/mL stock concentration) was evaporated under a stream of nitrogen. First, 1 μL PBS and then 180 μL extracted GPMVs were added onto the dried Ni-NTA lipid. After vortexing for 10 s, 65° heat was applied for 5 min, followed by cooling for 10 min to RT. The mixture was then left to sit at RT for 20 min before labeling. In phase-separated GPMVs, due to the high concentration of DTT treatment to induce phase separation, the nickel on the lipid was likely reduced and sometimes formed a brownish pellet at the bottom of the tube. However, the formation of pellets from the Ni-NTA lipid was not the issue for GPMVs prepared from CHO cells, mainly because of the 10 times less concentrated DTT treatment during its extraction.

For labeling both GUVs and GPMVs, AbStR-DPPE at a concentration of 0.25 μg/mL was added into the vesicle solution and incubated for 15 to 30 min to allow its integration into the membrane. Samples were further supplemented with different A488-labeled ECDs with a final concentration of 0.5 μg/mL and incubated for 15 to 30 min for His-Ni-NTA coupling. To reduce the background signal by unbound proteins, the labeled GUVs can be subsequently washed twice with 750 μL PBS, followed by allowing sedimentation for 20 to 30 min after each wash. The 80–100 μL GUVs from the bottom of the tube after sedimentation can then be used for measurements. The confocal imaging was performed in Ibidi 8-well plastic bottom chambers, while Ibidi 8- or 18-well glass-bottom chambers (#1.5H) were used for the FCS measurement. The wells were pretreated with 1 mg/mL bovine serum albumin beforehand for at least 1 h and washed three times with PBS. All pipetting was performed gently, and pipette tips were cut to reduce shear stress on the vesicles.

Confocal imaging of phase-separated GUVs

The Zeiss LSM 780/980 confocal laser scanning microscope equipped with a 40 × 1.2 water-immersion objective was used for imaging and FCS experiments. The microscope's objective was additionally provided with a DIC Prism (DIC Prism III PA 63×/1.40, model 426,957, Zeiss, Jena, Germany) for excitation laser depolarization for imaging experiments. Green and far-red fluorescence were excited with 488 and 633 nm lasers, and their fluorescent emission was detected in two channels of the 32-channel detector within the spectral windows of 498–580 and 641–696 nm, respectively. The pinhole parameter was set to 1 Airy unit. The dynamic range was 12 bits (4,096 gray value units), with four times line averaging. The confocal image was taken by positioning the focal plane at the GUV equatorial plane. Line plot profile of fluorescence intensity was performed using ImageJ (49). Fluorescence intensities in L_d and L_o phases were calculated from the plot peaks. Two directly opposite sides were chosen in the vesicles to eliminate the light intensity difference due to laser polarization. L_o partitioning (%L_o) was calculated by dividing the fluorescence intensity of L_o by the sum of the fluorescence intensities of L_o and L_d.

FCS measurement

The calibration of the pinhole position and the correction collar of the objective for FCS was performed before the measurement using Alexa 488-Alexa 647 solution mixture in water in the same Ibidi chamber as the samples. For FCS in GUVs or GPMVs, the focal plane was positioned at the bottom or the top of the vesicle, respectively. FCS curves were obtained for 5–10 s with low laser power (2–5 microwatts) with 5–10 repetitions to prevent photobleaching. The obtained correlation curves were fit and analyzed by FoCuS-point software (50).

ECD conformation prediction

The three-dimensional conformation of the ECDs were in situ predicted via AlphaFold (AlphaFold2_advanced.ipynb, collabfold). The details of the parameter are shown in Table S2, and the amino acid sequences are shown in Table S3. These structures were subsequently aligned in UCSF ChimeraX (v.1.3) with published AlphaFold predictions of the full-length proteins to assess the folding of the isolated ECDs.

Statistical analysis

The statistical analysis of each data set is explained in the corresponding figure legends.

RESULTS AND DISCUSSION

We used GUVs and GPMVs as model systems to mimic cellular membrane surfaces and study protein behavior. The main advantage of GUVs is finely controlled lipid composition while that of GPMVs is the near-native composition. We studied protein phase partitioning in phase-separated GUVs and protein diffusion in GUVs and GPMVs.

Saturated and unsaturated nickel-chelating lipids partition into L_o and L_d phase, respectively

An important dynamic behavior of proteins is their partitioning to certain lipid environments. To study the role of ECD size on protein partitioning, we first prepared phase-separated GUVs (DOPC/SM/Chol, 2:2:1) with 1 mol % nickel-chelating lipid that can directly bind to 6xHis-tagged ECDs (Fig. 1 *A*). To visualize phase separation, we used AbStR-DPPE that partitions preferentially to L_d (Fig. 1 *A* and *B*). To evaluate the partitioning of proteins in both ordered and disordered phases, we used two different nickel-chelating lipids: unsaturated 18:1/18:1 (we will refer to it as di-oleyl [DO] Ni-NTA) and saturated 16:0/16:0 Ni-NTA (we will refer to it as di-palmitoyl [DP] Ni-NTA). To recognize and characterize different Ni-NTA lipids, we used A488-6xHis. In GUVs with DO Ni-NTA, A488-6xHis preferentially binds to L_d, and in GUVs with DP Ni-NTA, it is preferentially associated with L_o (Fig. 1 *B*).

Artificial phase domains in GUVs display high contrast in membrane order between L_o and L_d domains. Alternatively, cell-derived GPMVs present more complex domains with a less dramatic difference in membrane order. To evaluate the partitioning of these Ni-NTA lipids in a more natural system, we incorporated nickel-chelating lipids into GPMVs (Fig. 1 *C*). However, incorporating Ni-NTA lipids to GPMVs is rather challenging compared with GUVs, presumably due to the charged surface of GPMVs (as the negatively charged lipids flip from the inner leaflet). Another possible reason might be the high DTT concentration used to form phase-separated GPMVs, which possibly reacts with nickel and reduces it. Despite these challenges, we obtained a small number of phase-separated GPMVs with nickel-chelating lipids attached to A488-6xHis and confirmed that DO Ni-NTA partitions to the disordered domains, whereas DP Ni-NTA prefers the ordered domains (Fig. 1 *D*).

Since GUVs with Ni-NTA lipids were significantly easier to prepare, we continued the rest of the partitioning experiments using GUVs, where we evaluated the role of ECD size on partitioning. For this purpose, we selected ECDs of proteins mainly involved in immune signaling, CD59, CD2, ICAM-1, and CD45, whereas A488-6xHis was used as a control (Fig. 1 *E*). Predicted (P) and apparent (A) molecular mass of these proteins are shown in Fig. 1

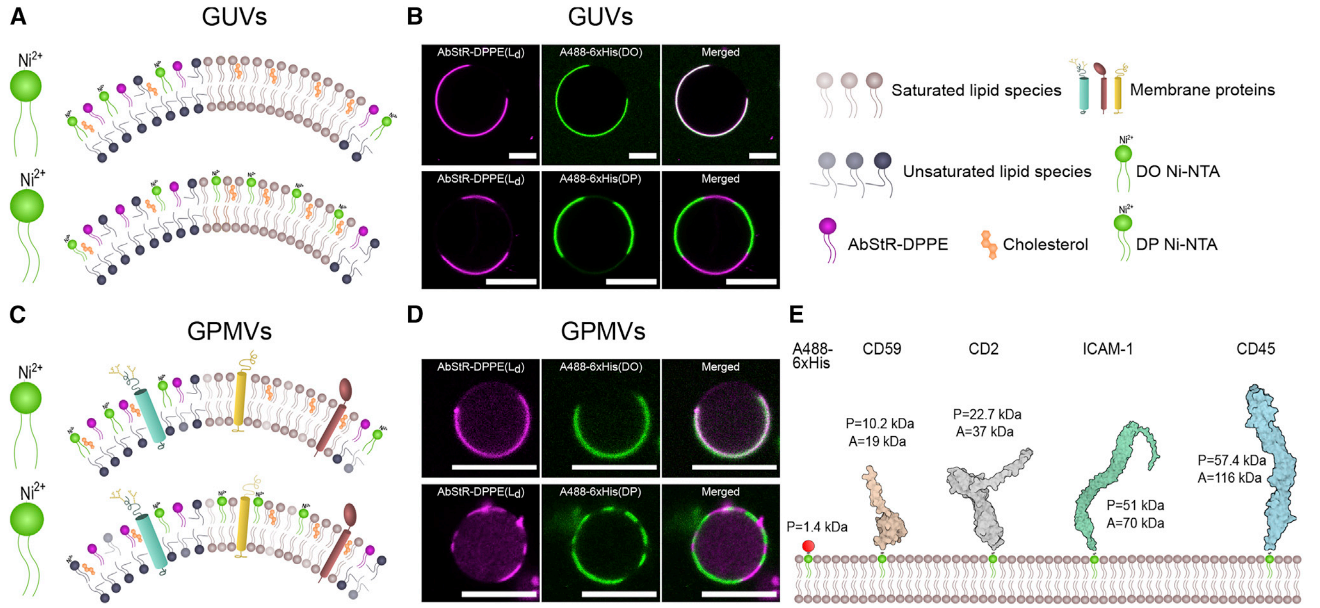

FIGURE 1 Ni-NTA lipid constituted biomimetic membranes with His-tagged proteins. (*A* and *B*) Cartoon (*A*) and confocal microscopy (*B*) images of phase-separated GUVs incorporated with DO and DP Ni-NTA. GUVs were composed of the ternary mixture of DOPC/SM/Chol (2:2:1). (*C* and *D*) Cartoon (*C*) and confocal microscopy (*D*) images of phase-separated GPMVs incorporated with DO and DP Ni-NTA. GPMVs were extracted from HeLa cells. AbStR-DPPE (*magenta*) and DO Ni-NTA incorporate preferentially in the disordered phase, whereas DP Ni-NTA prefers the ordered phase. Scale bars are 10 μm. (*E*) Proteins of interest used in the study (sizes are representative based on the structures). It shows the predicted (P) and apparent (A) mass of the proteins based on amino acids sequence and an SDS page, respectively. To see this figure in color, go online.

E, and the number of amino acids in these peptides are shown in Table S1. P and A are different primarily due to the glycosylation of the proteins. P is the molecular weight calculated using the amino acid sequence solely, while A is measured in an SDS page under reducing conditions, taking glycosylation of the protein into account. A is larger than P for all the proteins we used (Fig. 1 *E*) since they are produced in HEK293 cells and preserve native mammalian glycosylation patterns. The difference between A and P shows the level of glycosylation; the larger the difference, the more glycosylation the protein has.

ECD size influences domain partitioning

ECDs do not often directly interact with the PM. However, given the recent evidence on the impact of extracellular protein organization on membrane domains (22–25), ECDs might play a role in protein compartmentalization in the membrane. Earlier work using detergent resistance suggested that certain sequences in ECDs can be ordered domain targeting sequences (51–53). However, the size of the ECDs has not been evaluated in this context. Therefore, we set out to evaluate whether ECD size affects protein partitioning in phase-separated GUVs. To investigate this, ECDs of different sizes (Fig. 1 *E*) were incorporated into phase-separated GUVs (Fig. 2). The fluorescence intensity of the two peaks in the line profile across the GUV image (A488-protein and AbStR-DPPE) was used to calculate the percentage of L_o partitioning (Fig. 2 *A*).

To examine whether the concentration of the proteins on the surface affects partitioning, we first performed a control experiment with varying protein concentrations within the ranges we used (0.5 up to 2 μg/mL). The result indicates no effect of the concentration of the largest protein on its partitioning preference within the investigated concentration range (Fig. S1). It is worth noting that, at higher concentrations, protein crowding is likely to play a role; however, we always used a low concentration of proteins for our study, avoiding the impact of crowding effects on our investigation.

We next investigated the effect of ECD size on protein partitioning. As mentioned before, ECD size can be estimated using the amino acid sequence (P molecular mass), which ignores the posttranslational modifications such as glycosylation. Since many MPs are highly glycosylated, the A molecular mass is generally higher when measured under reduced conditions. Therefore, we first measured the partitioning as a function of A molecular mass; however, the P

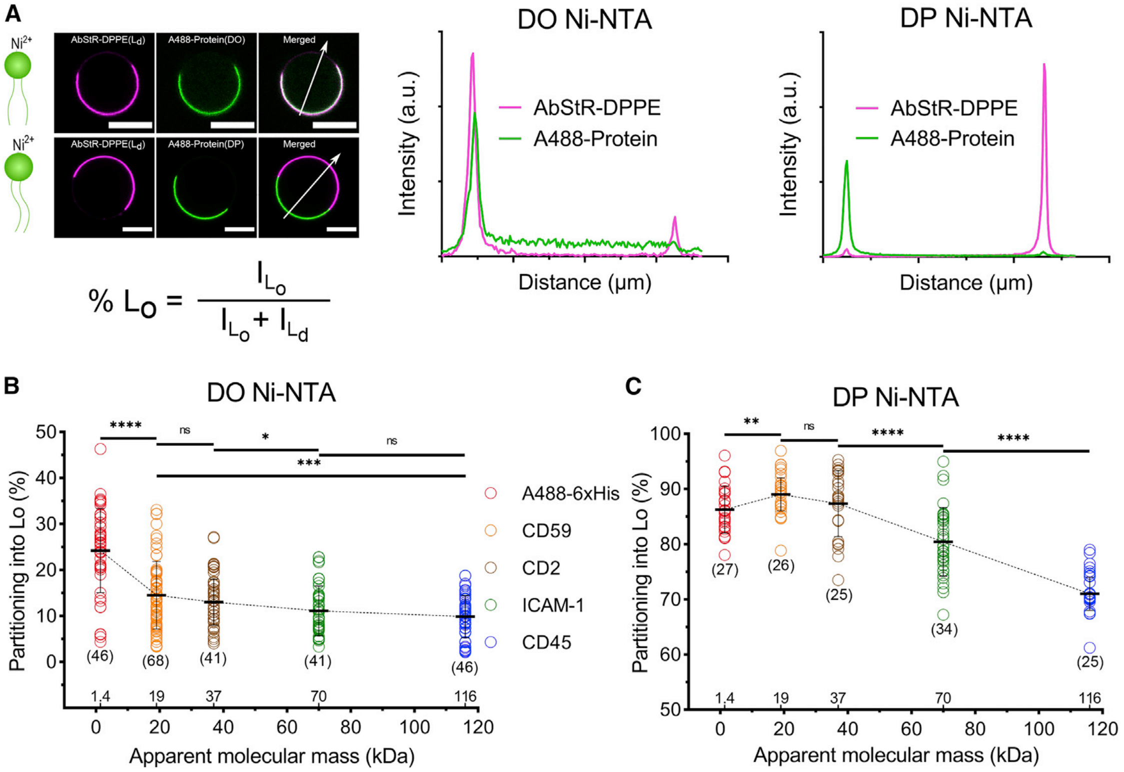

FIGURE 2 ECD mass plays a role in the protein partitioning in GUVs. (*A*) Line profile of fluorescence intensity across the GUVs indicated by the white arrow in the confocal images was used to calculate L_o percentage with the equation shown below the images. Scale bars are 10 μm. (*B*) L_o partitioning of different ECDs attached to DO Ni-NTA in GUVs. Student's *t*-test (two-tailed, nonparameterized) was used to determine significance (****$p < 0.0001$; ***$p = 0.0008$; *$p = 0.0393$; ns, nonsignificant). (*C*) L_o partitioning of the different ECDs attached to the DP Ni-NTA lipid in GUVs. Student's *t*-test (two-tailed, nonparameterized) was used to determine significance (**$p = 0.005$). Horizontal lines show the mean, and error bars represent the standard deviation. The numbers of data points obtained from experiments are indicated on the graphs in parentheses. To see this figure in color, go online.

mass and A mass for these proteins show the same increasing trend, i.e., as the P mass increases, the A mass also increases for the chosen proteins. As expected, in GUVs with DO Ni-NTA, proteins partitioned preferentially to L_d, whereas in GUVs with DP Ni-NTA, they partitioned to L_o. Interestingly, in both systems, L_o partitioning decreased as the A molecular mass increased (Fig. 2 *B* and *C*). These findings imply that A ECD mass is one of the determining factors for protein partitioning, and larger (and potentially highly glycosylated) proteins might be less abundant in ordered domains. This could be explained by different scenarios that need to be addressed. First, we needed to rule out any direct and differential interactions between different ECDs and the membrane. To this end, we generated the structures of the proteins via AlphaFold (Fig. S2; Tables S2 and S3) and measured the propensity of different proteins to interact with the membrane (Fig. S3) (54). This analysis showed that the hydrophobicity of the proteins is very similar, and there are not any notable potential interactions between the membrane and the ECDs. Therefore, the differences in hydrophobicity or membrane interactions cannot be responsible for the size-dependent partitioning we observed. Clustering might be another possible reason for different partitioning. To test whether our proteins clustered differently, we measured the apparent brightness of each protein with FCS. Apparent brightness for proteins was random (i.e., did not follow any trend with the size of the protein) and likely determined by the number of conjugatable amino acid moieties in the ECDs (Fig. S4). To be able to account for the reason behind the discrepancy in the partitioning of ECDs of different sizes, we needed to add more proteins into our measurements. While the proteins we tested above have a clear trend in A mass correlated with their partitioning, they also have a similar trend in P mass. To pinpoint the mechanism causing the difference in partitioning, we tested three more proteins with different trends in P versus A mass.

ECD glycosylation influences domain partitioning

Glycosylation is a posttranslational modification, leading to an addition of carbohydrates on proteins. It makes the proteins more rigid and heavier such that highly glycosylated proteins can be a few times heavier than their P mass (from their amino acid sequence). To test how the discrepancy between P and A mass affects the partitioning of ECDs, we picked three proteins from the same family with different degrees of glycosylation: CD34, PODXL, and PODXL2 (Fig. 3 *A*). PODXL is the most heavily glycosylated form, followed by PODXL2 and CD34. Interestingly, the P mass of PODXL is lower than PODXL2, but due to glycosylation, the A mass of PODXL becomes significantly higher than PODXL2. Therefore, the partitioning behavior of these three proteins will show whether A or P mass determines the partitioning. When we compared their partitioning in GUVs, we observed a clear trend between partitioning and the A mass but not the P mass (Fig. 3 *B* and *C*). To further confirm the role of glycosylation in partitioning, we removed the sugar residues from the proteins by a deglycosylation enzyme mixture (55). After the enzyme treatment, we observed an increase in the partitioning of proteins into L_o, except CD34, confirming the role of glycosylation in partitioning (Fig. 3 *D*). CD34 is the least glycosylated of these three proteins, therefore it is expected to see the smallest difference for CD34 upon deglycosylation. However, seeing no difference is presumably due to the incomplete effect of the enzyme on different glycosylation patterns (N- or O-glycosylation or sialylation).

Our data align with some previous reports while partially contradicting some others. For example, Rubio-Sánchez et al. showed size-dependent partitioning of oligonucleotides in model membranes where larger oligonucleotides partitioned less into the ordered domains, which is in line with our observations with proteins (27). However, by using detergent resistance assay, Shao et al. showed that O-glycans direct some adhesion molecules to ordered membrane domains, which contrasts with our findings (53). This discrepancy might be due to the different experimental systems (detergent resistance versus model membranes) or investigated glycosylation patterns (O-glycosylation versus overall glycosylation).

While our data could show that glycosylation and the A molecular mass (as we summarized with the term "size") are determinants of partitioning, the exact physical mechanism is not trivial to unravel since we do not have sufficient information on the exact geometry of the reconstituted proteins on membranes such as rigidity, bulkiness, length, apparent radius, and bending. Therefore, we cannot use such parameters in a quantitative model. However, a plausible speculation would be the excluded volume interactions: larger, glycosylated, and charged (due to sialic acid) molecules might repulse each other from tightly packed ordered domains (since lipid per area, hence molecular proximity, is higher in L_o phase), which might account for their exclusion from ordered domains. This effect can be attributed to A molecular mass but also to other properties such as charge, rigidity, bulkiness, length, or apparent radius of the molecule. With our current knowledge, we have the exact information about the A molecular mass of the proteins, which is perfectly in trend with observed partitioning for the proteins we used. However, the other factors might better account for the observed effects, which need to be addressed in the future with more experimental and in silico work.

ECD mass and glycosylation influence molecular diffusion

Another dynamic behavior of proteins is their diffusion as it determines their interaction dynamics with other macromolecules. There is still a controversy about the structural determinants of MP mobility. From the very first diffusion model (35) to the succeeding modifications (38–42), none of them

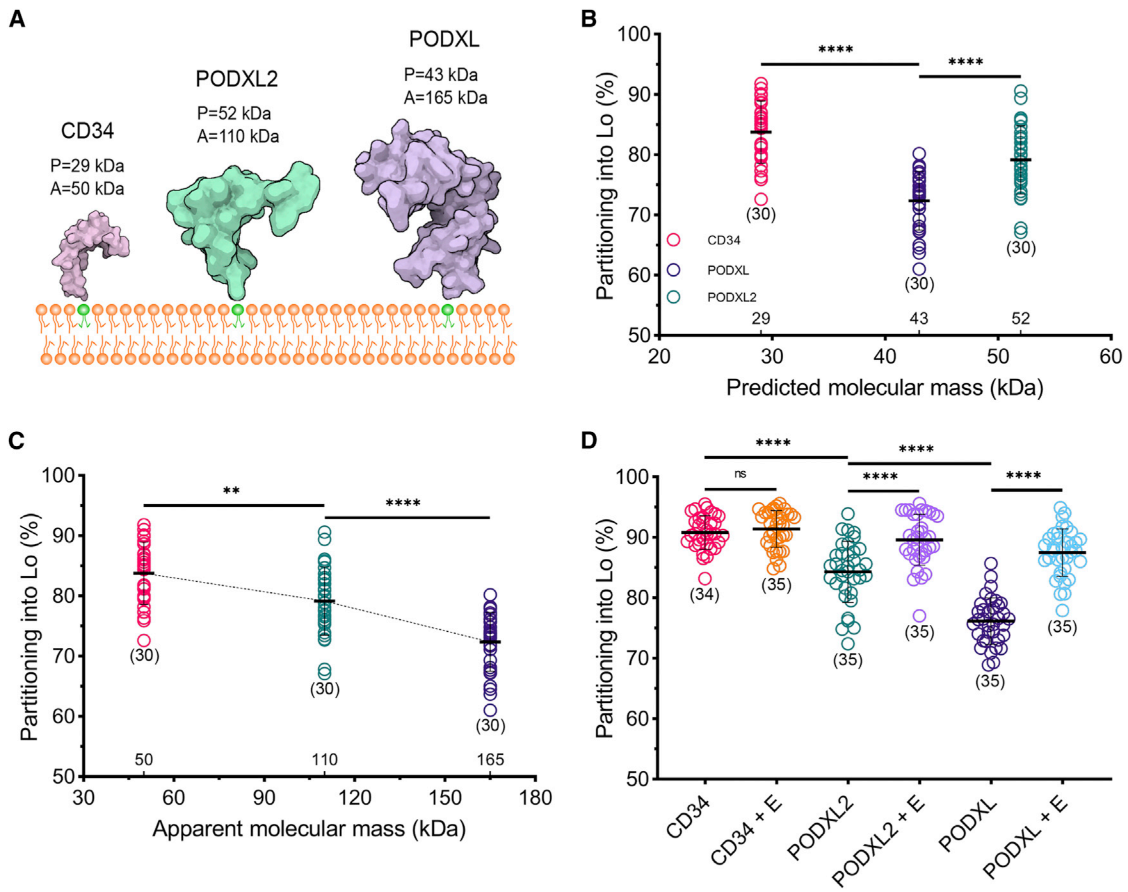

FIGURE 3 Glycosylation and apparent ECD mass play a role in protein partitioning in GUVs. (*A*) Cartoon of CD34, PODXL, and PODXL2 (sizes are representative based on the structures). (*B*) L_o partitioning of different ECDs attached to DP Ni-NTA in GUVs as a function of the predicted mass. Student's *t*-test (two-tailed, nonparameterized) was used to determine significance (****p $<$ 0.0001). (*C*) L_o partitioning of the different ECDs attached to the DP Ni-NTA lipid in GUVs as a function of the apparent mass. Student's *t*-test (two-tailed, nonparameterized) was used to determine significance (**p = 0.0028). (*D*) L_o partitioning of the different ECDs after the deglycosylation enzyme treatment (+E). Student's *t*-test (two-tailed, nonparameterized) was used to determine significance (ns, nonsignificant). Horizontal lines show the mean, and error bars represent the standard deviation. The numbers of data points obtained from experiments are indicated on the graphs in parentheses. To see this figure in color, go online.

explicitly take ECD size into account. There is a consensus in the field that protein diffusion in the membrane is determined by the TMD, the lipid environment, and their attachment to the membrane. To investigate the effect of ECDs on protein dynamics in the membrane, we measured the diffusion coefficients of proteins using FCS, where fluorescence intensity fluctuations were used to obtain information about molecular diffusion (Fig. 4 *A*). We first tested how the diffusion of ECDs was affected by their A mass in GUVs. For this purpose, we measured the diffusion of ECDs in POPC doped with 1 mol % DO Ni-NTA, and we observed a reciprocal relationship between the mobility and the ECD mass, that is, an increase in the size of the protein leads to slower diffusion (Fig. 4 *B*). The same relationship was observed in GUVs of different lipid compositions as well (Fig. S5). The diffusion of the lipid analog AbStR-DPPE displays no change in GUVs with ECDs of different sizes (Fig. S6), confirming that the membrane physical parameters (e.g., fluidity) and the experimental conditions (e.g., temperature fluctuations), did not cause the differences in diffusion for different ECDs. Moreover, the number of particles in each experimental setup for all proteins was comparable, ruling out the effect of crowding (46) on observed differences in diffusion (Fig. S7). To evaluate the role of glycosylation and A versus P mass on diffusion, as we did for partitioning, we used CD34, PODXL, and PODXL2. Similar to partitioning, we observed a correlation between diffusion and A mass but not with P mass (Figs. 4 *C* and *D* and S8–S10; Tables S2 and S3). When we treated these proteins with deglycosylation enzymes, PODXL2 and PODXL sped up significantly, while CD34 did not change notably, similar to partitioning experiments (Figs. 4 *E* and S11).

Our findings support some of the recent reports (45,47,48) while partially contradicting some others (21,46). For example, Houser et al. found size-dependent diffusion only in crowded conditions, while we observed this phenomenon in noncrowded conditions as well (46). The likely cause of this could be a different model membrane system (supported

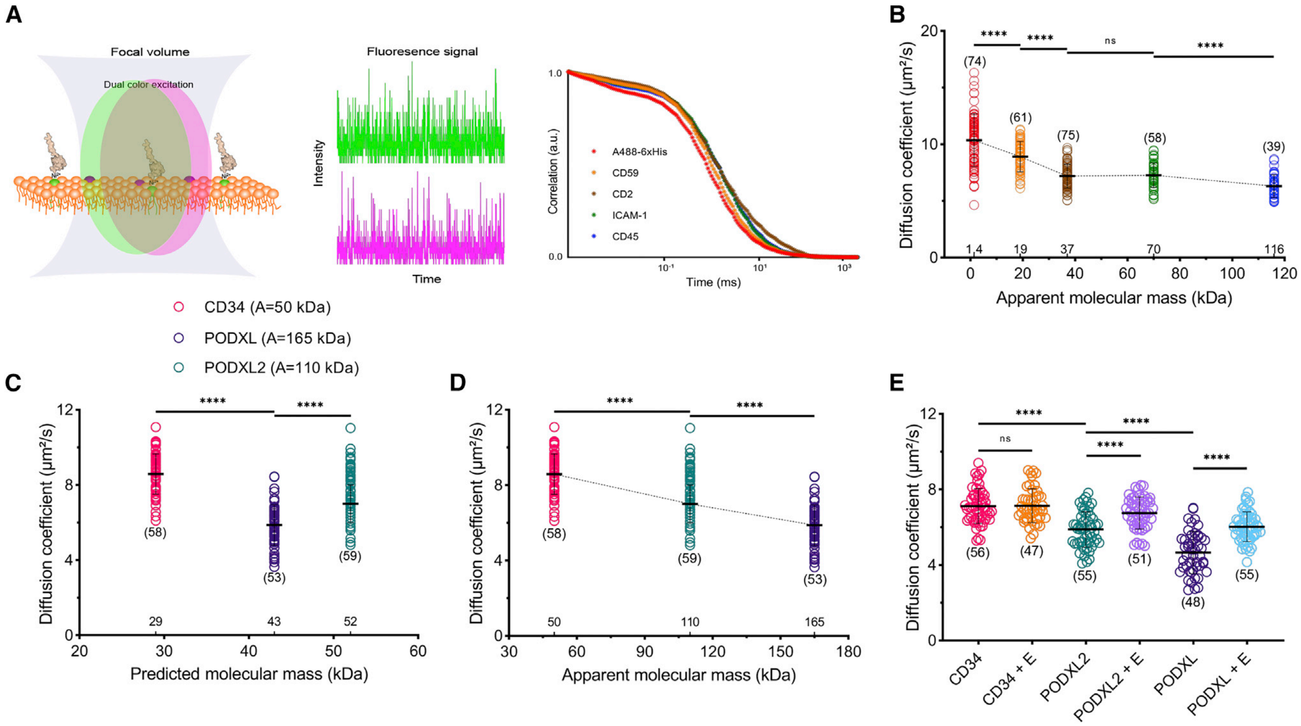

FIGURE 4 Apparent ECD mass and glycosylation determine the protein diffusion in GUVs. (*A*) Illustration of the FCS experiment. (*B*) Diffusion coefficients of the different ECDs in GUVs consisting of POPC. Student's *t*-test (two-tailed, nonparameterized) was used to determine significance (****p $<$ 0.0001; ns, nonsignificant). (*C* and *D*) Diffusion coefficients of CD34, PODXL, and PODXL2 (*C*) as a function of the predicted mass and (*D*) as a function of the apparent mass. (*E*) Diffusion coefficients of CD34, PODXL, and PODXL2 after deglycosylation enzyme treatment. Horizontal lines show the mean, and error bars represent the standard deviation. The numbers of data points obtained from experiments are indicated on the graphs in parentheses. To see this figure in color, go online.

bilayers versus free-standing membranes). Moreover, Popik et al. did not find glycosylation as a factor changing the ordered domain localization, while our data show a clear dependence of partitioning on the glycosylation of the proteins (21). This could be due to discrepancies of experimental modalities (e.g., detergent resistance assay versus model membranes). Our results are complementary to those of Hartel et al., as they investigate the impact of size extension/truncation of the pathogen GPI-anchored VSG glycoprotein (45). They reveal a correlation between diffusion and the ectodomain size, which can be different for different variants of the protein. With our study, we can extend the finding of size-dependent mobility to PM residing proteins in a more inclusive approach.

GPMV membrane environment reduces the size effect on molecular mobility

The cellular PM is a more crowded and complex environment than GUVs. The size effect on diffusion can be enhanced or diluted in crowded environments. For example, Houser et al. showed that in crowded environments, the size of the proteins plays a greater role in their diffusion compared with noncrowded environments (46). To test how our proteins behave in near-physiological crowding, we performed diffusion experiments on GPMVs (Fig. 5 *A*). The variation in membrane compositions between individual GPMVs leads to larger variability of the diffusion coefficient, therefore the data spread in graphs is larger (Fig. 5 *B*). Despite the large standard deviations, diffusion of proteins in GPMVs generally resembles the trend in GUVs, with certain exceptions. The diffusion coefficient decreases with an increase in ECD size for the first three small molecules. For relatively larger proteins, diffusion did not differ drastically. AbStR-DPPE shows no significant change in diffusion with ECDs of different sizes, confirming that the observed differences are indeed due to ECD size (Fig. S12). These data mean that in GPMVs, the size of ECDs is important, but in contrast to GUVs, small differences in size can be masked by other factors in the native membranes such as molecular crowding. However, additional factors in GPMVs might be responsible for this effect. For example, GPMVs exhibit negatively charged lipids in the outer leaflet, which ECDs can interact with. In contrast, in live cells (or in GUVs), there are not negatively charged lipids exposed in the outer leaflet. Second, chemicals used for vesiculation can lead to unforeseen effects on protein mobility. Moreover, the incorporation of nickel-chelating lipids into GPMVs is a burdensome protocol that might change the GPMV membranes. Therefore, the

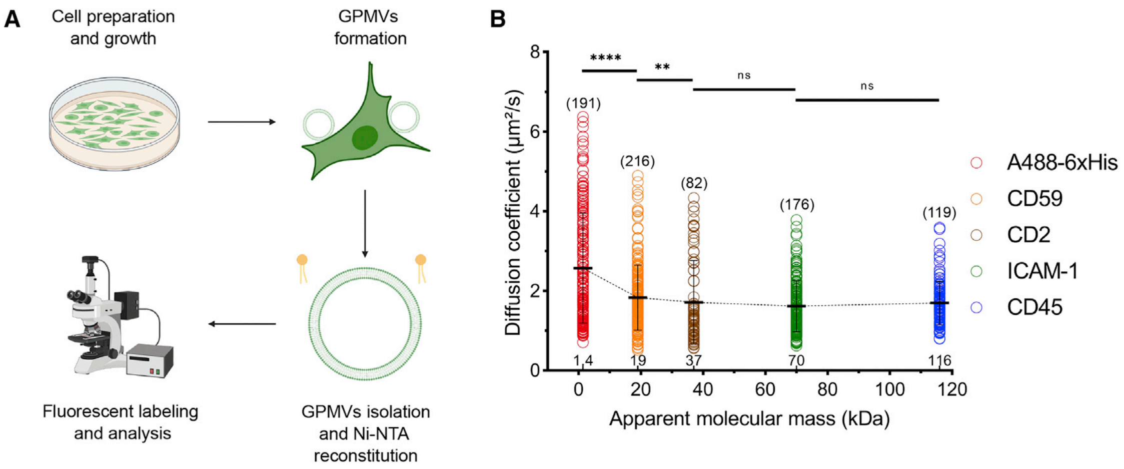

FIGURE 5 ECD mass and protein diffusion in GPMVs. (*A*) Preparation of GPMVs functionalized with Ni-NTA. (*B*) Diffusion of the different ECDs in GPMVs. Student's *t*-test (two-tailed, nonparameterized) was used to determine significance (****$p < 0.0001$; **$p = 0.0054$; ns, nonsignificant). Horizontal lines show the mean, and error bars represent the standard deviation. The numbers of data points obtained from experiments are indicated on the graphs in parentheses. To see this figure in color, go online.

results obtained from GUVs and GPMVs cannot be compared directly in the context of crowding. Yet, our results display an indication of possible effects of size in two different model membrane systems of varying membrane complexity.

CONCLUSION

In this study, we investigated the largely overlooked role of ECD size on MP dynamics. Our results show a decrease both in partitioning into ordered domains and in diffusion coefficient with the increase in ECD size. In light of our data, we suggest that ECD is a necessary component for understanding protein behavior in the PM. Our data highlight the necessity of new models explaining the diffusion and partitioning of proteins, taking their extracellular size into account. Accumulating experimental and in silico data will eventually lead to more refined and accurate models.

Our data have potential implications for the fundamental understanding of cellular biology. Differences in ECD size, for example, between isotypes or caused by posttranslational modifications, ligand binding, or enzymatic cleavage could lead to different protein mobility and localization in the PM, which could be required for certain functions. Therefore, ECD size might serve as a parameter for the compartmentalization of membrane components by influencing their mobility and domain partitioning. This postulation is in line with recent evidence on how glycosylation influences protein compartmentalization and cellular signaling (53,56,57). In this regard, our data can be the connection between different mechanisms proposed for MP reorganization during signaling initiation since they show the correlation between ECD size, diffusion, and domain partitioning.

There are currently multiple mechanisms proposed for T cell signaling (and most are applicable to other immune cell types). Size-, lipid-, or diffusion-dependent exclusion of inhibitory molecules (such as CD45) from the immune synapse are three of the main and seemingly distant mechanisms explaining T cell signaling (3–8). However, our results suggest that all three phenomena can be intertwined. The extracellular size, lipid domain partitioning, and molecular diffusion are not entirely independent. Tall and heavily glycosylated (hence more rigid) molecules can be driven out of a certain lipid environment and diffuse slower due to their size. Of course, size-dependent partitioning is unlikely to be the only mechanism determining whether immune cells will be activated or not; however, it would be a plausible fine-tuning mechanism. The exact role of size-dependent partitioning of proteins in immune signaling needs to be tested in the future. Similar to size-dependent partitioning, size-dependent diffusion of molecules can also fine-tune signaling output by determining the collision rate between the receptor and the activating and inhibitory proteins. The activating molecules for immune signaling (e.g., Lck kinase that phosphorylates the T cell receptors) are usually small proteins, while inhibitory molecules (e.g., CD45 phosphatase that dephosphorylates the T cell receptors) bear large ECDs. This discrepancy likely plays a role in their diffusion. Since diffusion rate is the currency for molecular interactions, size-dependent diffusion is a potential effector of immune signaling.

It is, however, essential to acknowledge the limitations of our study. The direct translatability from model membrane systems to the native PM requires caution. The absence of other MPs (i.e., absence of protein-protein interactions), the cortical actin cytoskeleton, and the extracellular networks (i.e., lack of crowding, extracellular matrix-protein interactions (34)) might hinder the direct translatability of GUV experiments. In addition, all ECDs but CD59 originate from

integral proteins, hence we refrain from drawing conclusions on full-length proteins. These questions can be studied in GPMVs bearing full-length proteins with the same TMDs but varying ECDs to be able to investigate these phenomena in a more physiological setting. However, GPMVs also have several shortcomings, such as lack of compositional control compared with GUVs and unpredicted effects of chemicals used for GPMV formation. Another possible caveat is the unpredictable geometry of ECDs (e.g., bending toward the membrane) due to the nature of the chemical coupling we used. However, previous studies confirmed that the height of the molecules is reasonably preserved when coupled to the membranes via His-Ni-NTA chemistry (58). Due to the sole focus on the ECD fraction of the proteins via membrane anchoring in this study, future validation experiments investigating the impact of the ECD in transmembrane proteins will strengthen our findings.

Despite the aforementioned limitations, our system provides a clean and controllable environment to evaluate how ECD size influences the dynamic behavior of the proteins independent of their TMDs or ICDs. We believe that this work will pave the way for further experiments and simulations, which will eventually lead to new models for protein dynamics in the PM.

SUPPORTING MATERIAL

Supporting material can be found online at https://doi.org/10.1016/j.bpj.2022.09.010. Data presented in this paper can be found online at: https://doi.org/10.17044/scilifelab.21102061.

AUTHOR CONTRIBUTIONS

All authors designed and performed research, C.O.G. and L.W. analyzed data, and all authors wrote the paper.

ACKNOWLEDGMENTS

We thank Hannes Feyrer and CSI:Nano Lab for their feedback on figures, Dr. Markus Deserno for discussing our results, and SciLifeLab Advanced Light Microscopy facility and National Microscopy Infrastructure (VR-RFI 2016-00968) for their support on imaging. C.O.G. was supported by a SciLifeLab summer internship (C-2021-0466). E.S. is supported by a Swedish Research Council Starting Grant (2020-02682) and by grants from Karolinska Institutet and the SciLifeLab National COVID-19 Research Program and is financed by the Knut and Alice Wallenberg Foundation. AlphaFold model analysis was performed via USCS ChimeraX developed by the Resource for Biocomputing, Visualization and Informatics at the University of California, San Francisco, with support from National Institutes of Health R01-GM129325 and the Office of Cyber Infrastructure and Computational Biology, National Institute of Allergy and Infectious Diseases. The figures were prepared by Inkscape and BioRender.

DECLARATION OF INTERESTS

The authors declare no competing interests.

REFERENCES

1. Fagerberg, L., K. Jonasson, ..., L. Berglund. 2010. Prediction of the human membrane proteome. *Proteomics.* 10:1141–1149.
2. Simons, K., and D. Toomre. 2000. Lipid rafts and signal transduction. *Nat. Rev. Mol. Cell Biol.* 1:31–39.
3. Davis, S. J., and P. A. van der Merwe. 2006. The kinetic-segregation model: TCR triggering and beyond. *Nat. Immunol.* 7:803–809.
4. Felce, J. H., E. Sezgin, ..., S. J. Davis. 2018. CD45 exclusion and cross-linking based receptor signaling together broaden FcεRI reactivity. *Sci. Signal.* 11:eaat0756.
5. Bakalar, M. H., A. M. Joffe, and D. A. Fletcher. 2018. Size-dependent segregation controls macrophage phagocytosis of antibody-opsonized targets. *Cell.* 174:131–142.e13.
6. Swamy, M., K. Beck-Garcia, ..., W. W. A. Schamel. 2016. A cholesterol-based allostery model of T cell receptor phosphorylation. *Immunity.* 44:1091–1101.
7. Chen, K. Y., E. Jenkins, ..., S. J. Davis. 2021. Trapping or slowing the diffusion of T cell receptors at close contacts initiates T cell signaling. *Proc. Natl. Acad. Sci. USA.* 118:e2024250118.
8. Urbančič, I., L. Schiffelers, ..., C. Eggeling. 2021. Aggregation and mobility of membrane proteins interplay with local lipid order in the plasma membrane of T cells. *FEBS Lett.* 595:2127–2146.
9. Sych, T., C. O. Gurdap, ..., E. Sezgin. 2021. How does liquid-liquid phase separation in model membranes reflect cell membrane heterogeneity? *Membranes.* 11:323.
10. Sezgin, E., I. Levental, ..., P. Schwille. 2012. Partitioning, diffusion, and ligand binding of raft lipid analogs in model and cellular plasma membranes. *Biochim. Biophys. Acta.* 1818:1777–1784.
11. Lorent, J. H., and I. Levental. 2015. Structural determinants of protein partitioning into ordered membrane domains and lipid rafts. *Chem. Phys. Lipids.* 192:23–32.
12. Polley, A., A. Orłowski, ..., I. Vattulainen. 2017. Glycosylation and lipids working in concert direct CD2 ectodomain orientation and presentation. *J. Phys. Chem. Lett.* 8:1060–1066.
13. Stone, M. B., S. A. Shelby, ..., S. L. Veatch. 2017. Protein sorting by lipid phase-like domains supports emergent signaling function in B lymphocyte plasma membranes. *Elife.* 6:e19891.
14. Yang, S.-T., A. J. B. Kreutzberger, ..., L. K. Tamm. 2017. HIV virions sense plasma membrane heterogeneity for cell entry. *Sci. Adv.* 3:e1700338.
15. Sharpe, H. J., T. J. Stevens, and S. Munro. 2010. A comprehensive comparison of transmembrane domains reveals organelle-specific properties. *Cell.* 142:158–169.
16. Diaz-Rohrer, B. B., K. R. Levental, ..., I. Levental. 2014. Membrane raft association is a determinant of plasma membrane localization. *Proc. Natl. Acad. Sci. USA.* 111:8500–8505.
17. Lorent, J. H., B. Diaz-Rohrer, ..., I. Levental. 2017. Structural determinants and functional consequences of protein affinity for membrane rafts. *Nat. Commun.* 8:1219.
18. Nalivaeva, N. N., and A. J. Turner. 2009. Lipid anchors to proteins. *In* Handbook of Neurochemistry and Molecular Neurobiology: Neural Lipids. A. Lajtha, G. Tettamanti, and G. Goracci, eds. Springer US, pp. 353–372.
19. van Deventer, S., A. B. Arp, and A. B. van Spriel. 2021. Dynamic plasma membrane organization: a complex symphony. *Trends Cell Biol.* 31:119–129.
20. Sezgin, E., I. Levental, ..., C. Eggeling. 2017. The mystery of membrane organization: composition, regulation and physiological relevance of lipid rafts. *Nat. Rev. Mol. Cell Biol.* 18:361–374.
21. Popik, W., and T. M. Alce. 2004. CD4 receptor localized to non-raft membrane microdomains supports HIV-1 entry: identification of a novel raft localization marker in CD4 *. *J. Biol. Chem.* 279:704–712.
22. Stachowiak, J. C., E. M. Schmid, ..., C. C. Hayden. 2012. Membrane bending by protein–protein crowding. *Nat. Cell Biol.* 14:944–949.

23. Yuan, F., H. Alimohamadi, …, J. C. Stachowiak. 2021. Membrane bending by protein phase separation. *Proc. Natl. Acad. Sci. USA.* 118:e2017435118.

24. Banjade, S., and M. K. Rosen. 2014. Phase transitions of multivalent proteins can promote clustering of membrane receptors. *Elife.* 3:e04123.

25. Zeno, W. F., K. E. Johnson, …, M. L. Longo. 2016. Dynamics of crowding-induced mixing in phase separated lipid bilayers. *J. Phys. Chem. B.* 120:11180–11190.

26. Varshney, P., V. Yadav, and N. Saini. 2016. Lipid rafts in immune signalling: current progress and future perspective. *Immunology.* 149:13–24.

27. Rubio-Sánchez, R., S. E. Barker, …, L. D. Michele. 2021. A modular, dynamic, DNA-based platform for regulating cargo distribution and transport between lipid domains. *Nano Lett.* 21:2800–2808.

28. Fujiwara, T., K. Ritchie, …, A. Kusumi. 2002. Phospholipids undergo hop diffusion in compartmentalized cell membrane. *J. Cell Biol.* 157:1071–1081.

29. Wawrezinieck, L., H. Rigneault, …, P. F. Lenne. 2005. Fluorescence correlation spectroscopy diffusion laws to probe the submicron cell membrane organization. *Biophys. J.* 89:4029–4042.

30. Lenne, P. F., L. Wawrezinieck, …, D. Marguet. 2006. Dynamic molecular confinement in the plasma membrane by microdomains and the cytoskeleton meshwork. *EMBO J.* 25:3245–3256.

31. Eggeling, C., C. Ringemann, …, S. W. Hell. 2009. Direct observation of the nanoscale dynamics of membrane lipids in a living cell. *Nature.* 457:1159–1162.

32. Alenghat, F. J., and D. E. Golan. 2013. Membrane protein dynamics and functional implications in mammalian cells. *Curr. Top. Membr.* 72:89–120.

33. Schneider, F., D. Waithe, …, E. Sezgin. 2017. Diffusion of lipids and GPI-anchored proteins in actin-free plasma membrane vesicles measured by STED-FCS. *Mol. Biol. Cell.* 28:1507–1518.

34. Frischknecht, R., M. Heine, …, E. D. Gundelfinger. 2009. Brain extracellular matrix affects AMPA receptor lateral mobility and short-term synaptic plasticity. *Nat. Neurosci.* 12:897–904.

35. Saffman, P. G., and M. Delbrück. 1975. Brownian motion in biological membranes. *Proc. Natl. Acad. Sci. USA.* 72:3111–3113.

36. Hughes, B. D., B. A. Pailthorpe, and L. R. White. 1981. The translational and rotational drag on a cylinder moving in a membrane. *J. Fluid Mech.* 110:349–372.

37. Guigas, G., and M. Weiss. 2006. Size-dependent diffusion of membrane inclusions. *Biophys. J.* 91:2393–2398.

38. Frick, M., K. Schmidt, and B. J. Nichols. 2007. Modulation of lateral diffusion in the plasma membrane by protein density. *Curr. Biol.* 17:462–467.

39. Petrov, E. P., and P. Schwille. 2008. Translational diffusion in lipid membranes beyond the saffman-delbrück approximation. *Biophys. J.* 94:L41–L43.

40. Ramadurai, S., A. Holt, …, B. Poolman. 2009. Lateral diffusion of membrane proteins. *J. Am. Chem. Soc.* 131:12650–12656.

41. Morozova, D., G. Guigas, and M. Weiss. 2011. Dynamic structure formation of peripheral membrane proteins. *PLoS Comput. Biol.* 7:e1002067.

42. Goose, J. E., and M. S. P. Sansom. 2013. Reduced lateral mobility of lipids and proteins in crowded membranes. *PLoS Comput. Biol.* 9:e1003033.

43. Weiß, K., A. Neef, …, J. Enderlein. 2013. Quantifying the diffusion of membrane proteins and peptides in black lipid membranes with 2-focus fluorescence correlation spectroscopy. *Biophys. J.* 105:455–462.

44. Wieser, S., M. Moertelmaier, …, G. J. Schütz. 2007. (Un)Confined diffusion of CD59 in the plasma membrane determined by high-resolution single molecule microscopy. *Biophys. J.* 92:3719–3728.

45. Hartel, A. J. W., M. Glogger, …, M. Engstler. 2015. The molecular size of the extra-membrane domain influences the diffusion of the GPI-anchored VSG on the trypanosome plasma membrane. *Sci. Rep.* 5:10394.

46. Houser, J. R., D. J. Busch, …, J. C. Stachowiak. 2016. The impact of physiological crowding on the diffusivity of membrane bound proteins. *Soft Matter.* 12:2127–2134.

47. Guigas, G., and M. Weiss. 2015. Membrane protein mobility depends on the length of extra-membrane domains and on the protein concentration. *Soft Matter.* 11:33–37.

48. Kim, D.-H., K. Zhou, …, S. H. Ryu. 2015. Analysis of interactions between the epidermal growth factor receptor and soluble ligands on the basis of single-molecule diffusivity in the membrane of living cells. *Angew. Chem., Int. Ed. Engl.* 54:7028–7032.

49. Schindelin, J., I. Arganda-Carreras, …, A. Cardona. 2012. Fiji: an open-source platform for biological-image analysis. *Nat. Methods.* 9:676–682.

50. Waithe, D., M. P. Clausen, …, C. Eggeling. 2016. FoCuS-point: software for STED fluorescence correlation and time-gated single photon counting. *Bioinformatics.* 32:958–960.

51. Yang, H., and E. L. Reinherz. 2001. Dynamic recruitment of human CD2 into lipid rafts. Linkage to T cell signal transduction. *J. Biol. Chem.* 276:18775–18785.

52. Yamabhai, M., and R. G. W. Anderson. 2002. Second cysteine-rich region of epidermal growth factor receptor contains targeting information for caveolae/rafts. *J. Biol. Chem.* 277:24843–24846.

53. Shao, B., T. Yago, …, R. P. McEver. 2015. O-glycans direct selectin ligands to lipid rafts on leukocytes. *Proc. Natl. Acad. Sci. USA.* 112:8661–8666.

54. Wang, L., J. Zhang, …, C. Song. 2022. Membrane contact probability: an essential and predictive character for the structural and functional studies of membrane proteins. *PLoS Comput. Biol.* 18:e1009972.

55. Lu, C.-H., K. Pedram, …, B. Cui. 2022. Membrane curvature regulates the spatial distribution of bulky glycoproteins. *Nat. Commun.* 13:3093.

56. Wasim, L., F. H. M. Buhari, …, B. Treanor. 2019. N-linked glycosylation regulates CD22 organization and function. *Front. Immunol.* 10:699.

57. Blouin, C. M., Y. Hamon, …, C. Lamaze. 2016. Glycosylation-dependent IFN-gammaR partitioning in lipid and actin nanodomains is critical for JAK activation. *Cell.* 166:920–934.

58. Son, S., S. C. Takatori, …, D. A. Fletcher. 2020. Molecular height measurement by cell surface optical profilometry (CSOP). *Proc. Natl. Acad. Sci. USA.* 117:14209–14219.

Biophysical Journal, Volume *121*

Supplemental information

Influence of the extracellular domain size on the dynamic behavior of membrane proteins

Cenk Onur Gurdap, Linda Wedemann, Taras Sych, and Erdinc Sezgin

Supplementary Data

Supplementary Figures

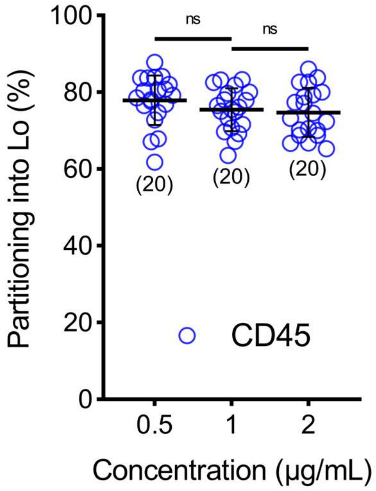

Supp. Fig. 1. The partitioning of CD45 (the largest protein) remains constant across varying protein concentrations. A non-parameterized ANOVA was used to determine significance (ns: non-significant). Horizontal lines show the mean and error bars represent the standard deviation. The number of data points obtained for each experiment are indicated on the graphs in parentheses.

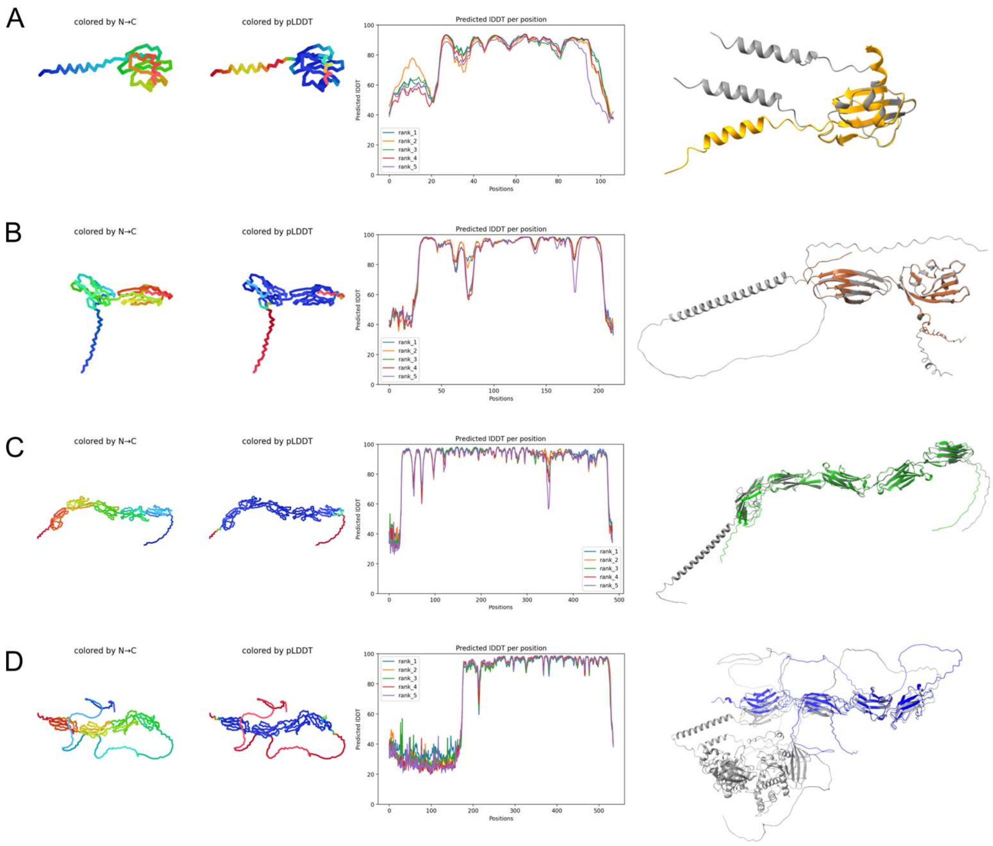

Supp. Fig. 2. ECD Structural models via ALPHAFOLD2. Model obtained via Collabfold, multiple sequence alignment performed via Jackhmmer, displayed is the highest ranked out of 5 models performed, with the LDDT graph for all models displayed next to the structures. Also shown are alignments to published structures for the whole protein from PDB, each being alphafold models, PDB code indicated with the protein as following: (A) CD59, AlphaFold: AF-P13987-F1, (B) CD2, AlphaFold: AF_P06729-F1, (C) ICAM-1, AlphaFold: AF-P05362-F1, (D) CD45, AlphaFold: AF-P08575-F1. Some of the structures cannot be resolved with Alphafold, presumably due to glycosylation.

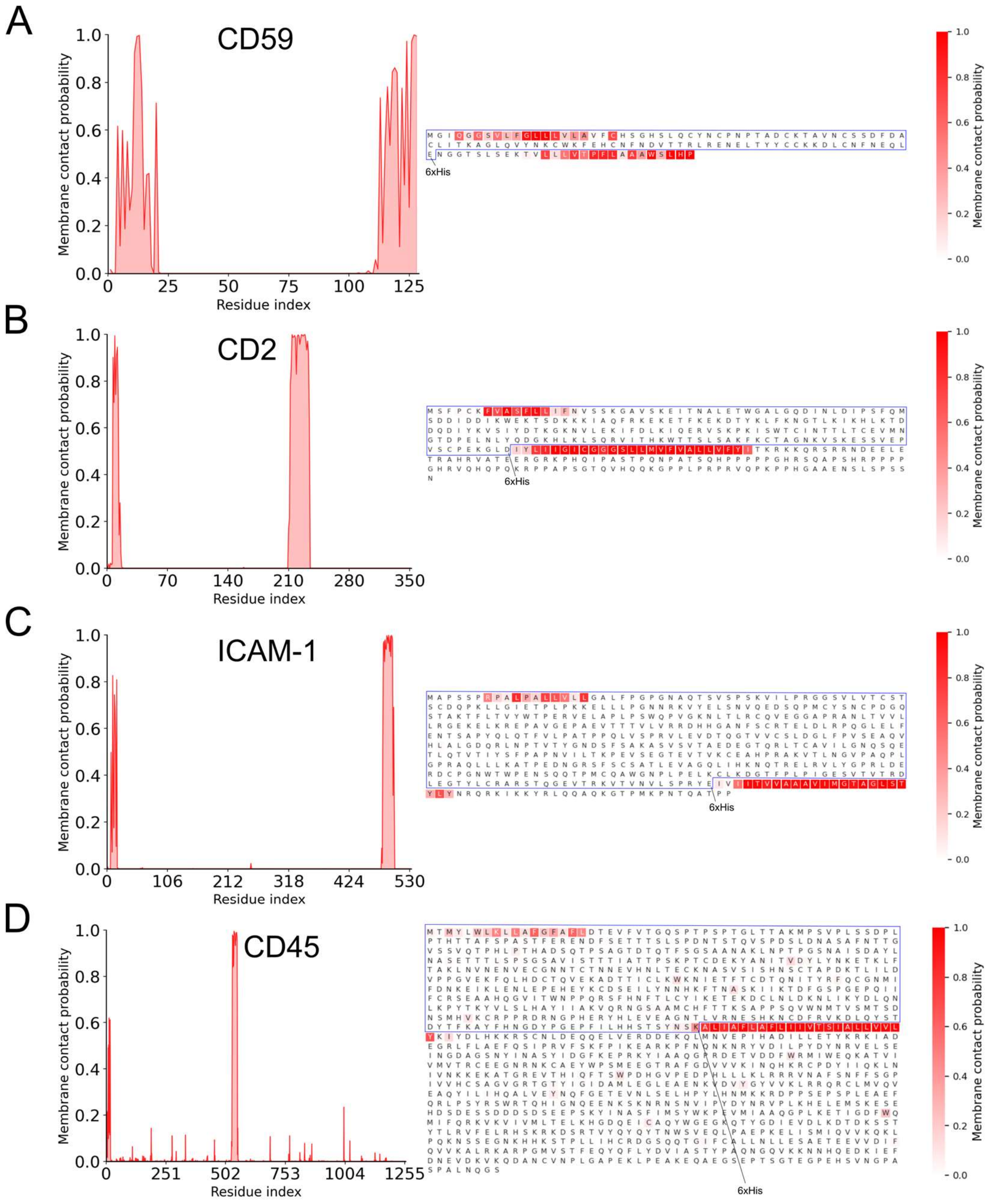

Supp. Fig. 3. Membrane contact probability. The analysis was done on full-length proteins and blue frames show extracellular domain of the proteins used in the study. 6xHis-tag in C-terminus is shown with arrow for each protein. Amino acids next the membrane anchoring sites can result in protein lipid interactions. Each protein has similar hydrophobic residues towards the far end of the proteins.

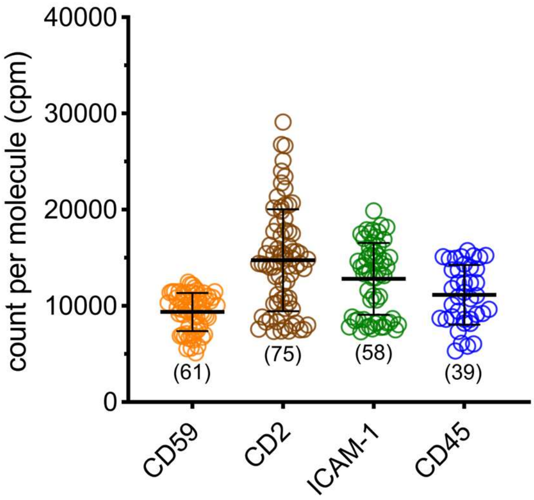

Supp. Fig. 4. Count per molecule for FCS measurement on GUVs. Horizontal lines show the mean and error bars represent the standard deviation. The number of data points obtained from experiments are indicated on the graphs in parentheses. Data does not show a trend that can correlate with diffusion or partitioning.

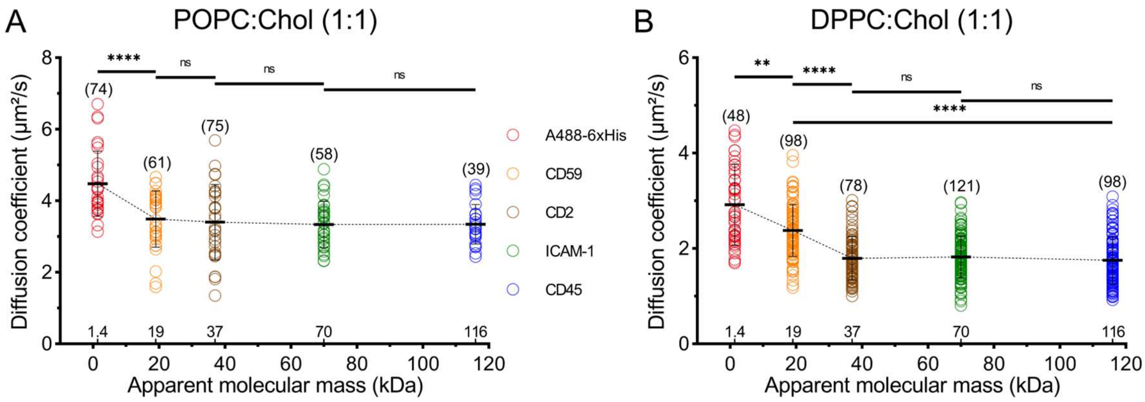

Supp. Fig. 5. Diffusion coefficients of the different ECDs in GUVs with (A) POPC:Chol (1:1) and (B) with DPPC/Chol (1:1). Student's t-test (two-tailed, non-parameterized) was used to determine significance (****P<0.0001; **P=0.001; ns: non-significant). Horizontal lines show the mean and error bars represent the standard deviation. The number of data points obtained from experiments are indicated on the graphs in parentheses.

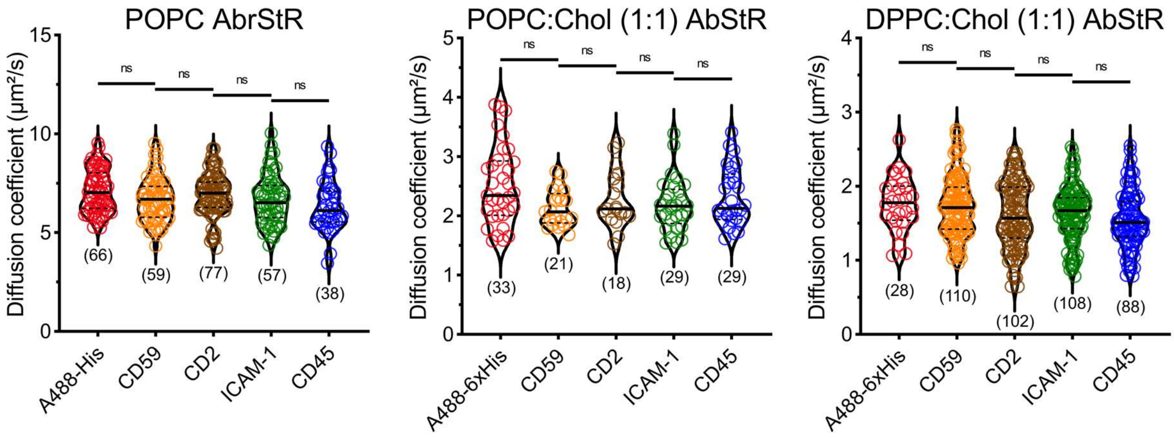

Supp. Fig. 6. Diffusion of membrane dye (AbStR-DPPE) in GUVs. Non-parameterized ANOVA was used to determine significance (ns: non-significant). Horizontal thick lines show the median and dotted lines represent the quartiles. The number of data points obtained from experiments are indicated on the graphs in parentheses.

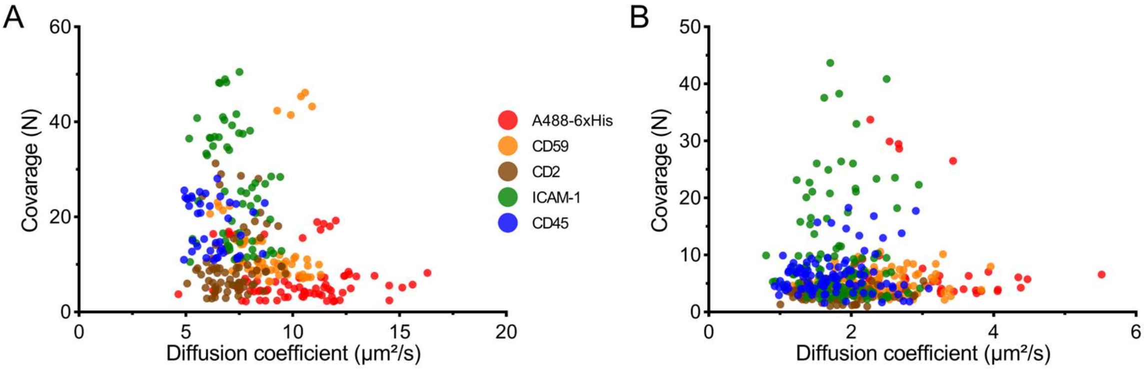

Supp Fig. 7. Number of molecules (coverage) vs diffusion time. Membrane coverage correlated to the diffusion coefficients obtained during the experiments for (A) POPC and (B) DPPC:Chol in GUVs. The plot shows no correlation between the mobility and membrane coverage, displaying that the experiments were performed under non-crowding affected conditions.

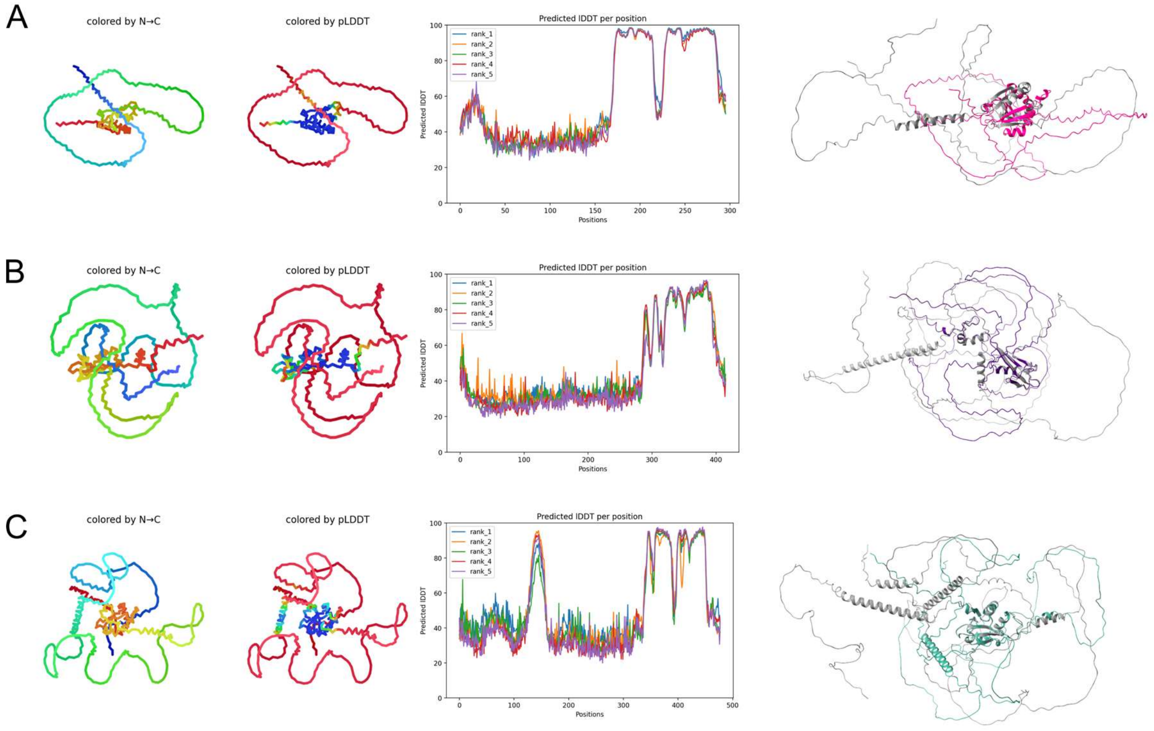

Supp. Fig. 8. ECD Structural models via ALPHAFOLD2. Model obtained via Collabfold, multiple sequence alignment performed via Jackhmmer, displayed is the highest ranked out of 5 models performed, with the LDDT graph for all models displayed next to the structures. Also shown are alignments to published structures for the whole protein's published Alphafold prediction. (A) CD34, AlphaFold: AF-P28906-F1, (A) CD34, AlphaFold: AF-P28906-F1, (B) PODXL, AlphaFold: AF-O00592-F1, (C) PODXL2, AlphaFold: AF-Q9NZ53-F1. Some of the structures cannot be resolved with Alphafold, presumably due to glycosylation.

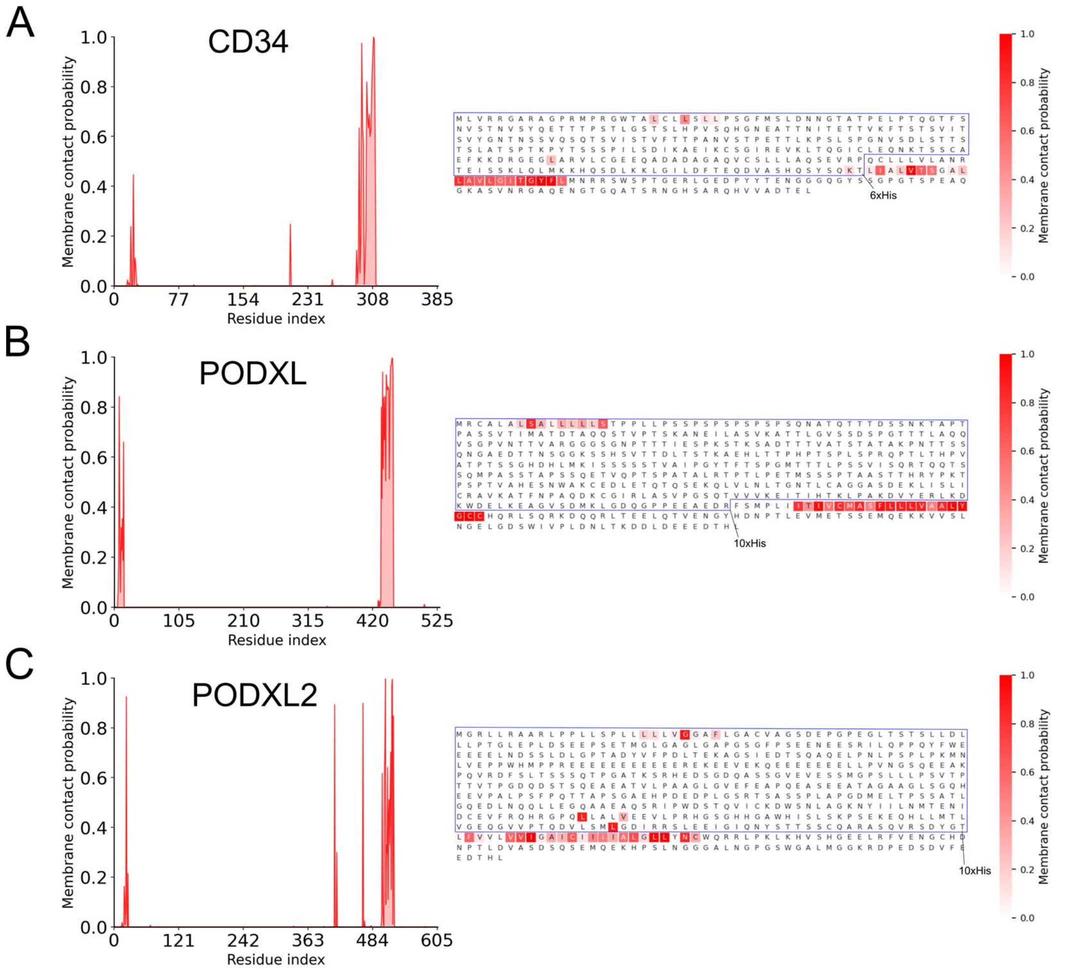

Supp. Fig. 9. Membrane contact probability. The analysis was done on the full-length proteins, and blue frames show extracellular domain of the proteins used in the study. The 6xHis-tag or 10xHis-tag at C-terminus is shown with arrow for each protein.

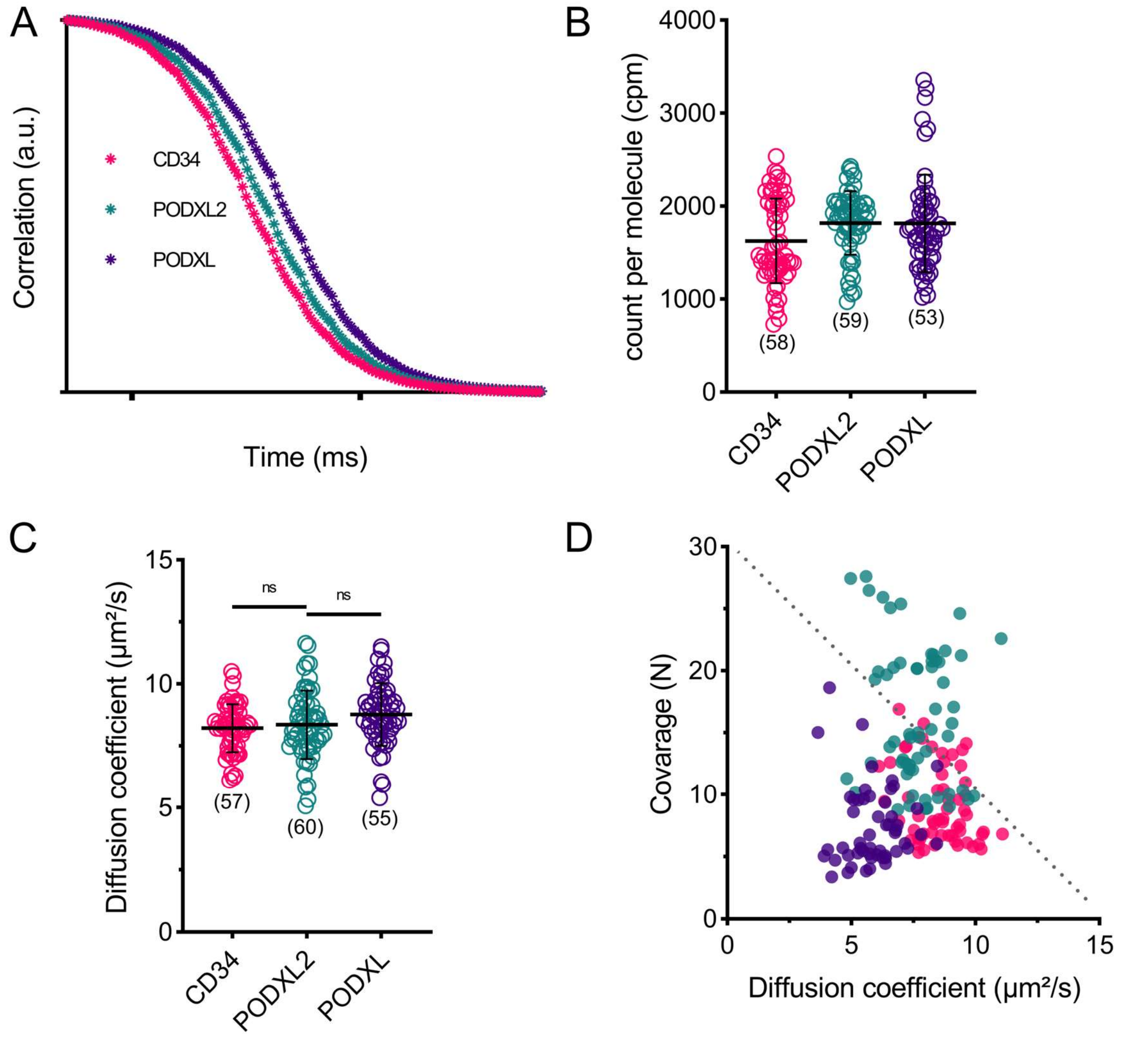

Supp. Fig. 10. Control experiments for CD34, PODXL2, and PODXL. (A) FCS curves. (B) Count per molecule for the FCS measurement in GUVs. (C) Diffusion of the membrane dye (AbStR-DPPE) in GUVs. (D) Number of molecules (coverage) vs diffusion time. Non-parameterized ANOVA was used to determine significance (ns: non-significant). Horizontal lines show the mean and error bars represent the standard deviation. The number of data points obtained from experiments are indicated on the graphs in parentheses.

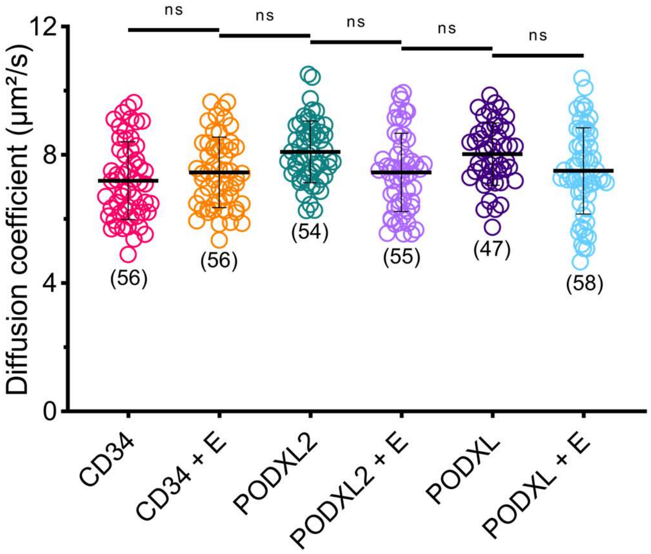

Supp. Fig. 11. Diffusion of membrane dye (AbStR-DPPE) in GUVs. Non-parameterized ANOVA was used to determine significance (ns: non-significant). Horizontal lines show the mean and error bars represent the standard deviation. The number of data points obtained from experiments are indicated on the graphs in parentheses. E stands for the enzyme treatment for the deglycosylation.

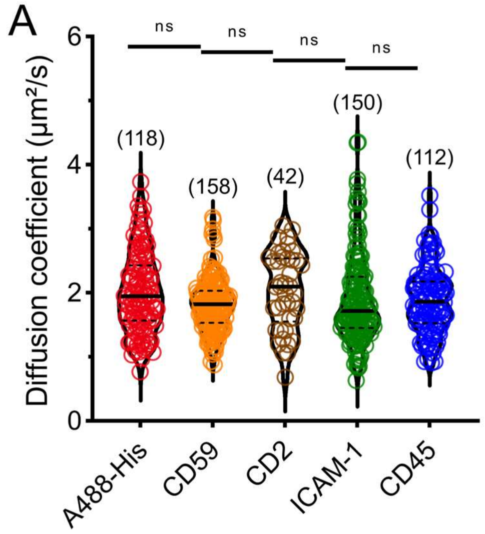

Supp. Fig. 12. Diffusion of membrane dye (AbStR-DPPE) in GPMVs. Non-parameterized ANOVA was used to determine significance (ns: non-significant). Horizontal thick lines show the median and dotted lines represent the quartiles. The number of data points obtained from experiments are indicated on the graphs in parentheses.

Supplementary Tables

Supp. Table 1: Molecular weight and required values for dye volume calculation for each of the ECDs investigated.

	Amino acids	MW_p (kDa)	ME	C_d (mg/mL)	V_d (µL)	Extinction coefficient ($M^{-1}cm^{-1}$)	C_p (mg/mL)
A488-6xHis	6	1.4	-	-	-	-	0.0025
CD59	101	19	3	5	1	12085	0.36
CD2	209	37	3	0.5	4	28210	0.32
ICAM-1	480	70	5	0.5	3	41785	0.43
CD45	529	116	5	0.5	3	51310	0.43
CD34	270	50	8	1	8.9	11835	~1
PODXL	405	165	8	1	6	15720	~1
PODXL2	468	110	8	1	5	35200	~0.8

Supp. Table 2: Alphafold2 (colabfold) parameters chosen during the model generation.

Parameter	Option chosen
**Search against genetic database**	
Msa_method	Jackhammer
Add_custom_msa	no
Pair_mode	Unpaired
Pair_cov	50
Pair-qid	20
**Filtering options**	
trim	no
Cov	no
Qid	no
**Run alphafold**	
Rank_by	pLDDT
Use turbo	Yes
Max_msa	512:1024
Num_models	5
Use_ptm	Yes
Num_ensemble	1
Max_recycles	3
Tol	o
Is_training	No
Num_samples	1
Subsample_msa	Yes
**Refine structures with Amber relax**	
Num_relax	None

Supp. Table 3: Amino acid sequences of the ECDs used in the project. Grey colors show the N-terminal 6xHis-Tag or 10xHis-tag.

ECD	Sequence
CD59	MGIQGGSVLFGLLLVLAVFCHSGHSLQCYNCPNPTADCKTAVNCSSDFDACLITKAGLQV<br>YNKCWKFEHCNFNDVTTRLRENELTYYCCKKDLCNFNEQLE HHHHHH
CD2	MSFPCKFVASFLLIFNVSSKGAVSKEITNALETWGALGQDINLDIPSFQMSDDIDDIKWE<br>KTSDKKKIAQFRKEKETFKEKDTYKLFKNGTLKIKHLKTDDQDIYKVSIYDTKGKNVLEK<br>IFDLKIQERVSKPKISWTCINTTLTCEVMNGTDPELNLYQDGKHLKLSQRVITHKWTTSL<br>SAKFKCTAGNKVSKESSVEPVSCPEKGLHHHHHH
Icam-1	MAPSSPRPALPALLVLLGALFPGPGNAQTSVSPSKVILPRGGSVLVTCSTSCDQPKLLGI<br>ETPLPKKELLLPGNNRKVYELSNVQEDSQPMCYSNCPDGQSTAKTFLTVYWTPERVELAP<br>LPSWQPVGKNLTLRCQVEGGAPRANLTVVLLRGEKELKREPAVGEPAEVTTTVLVRRDHH<br>GANFSCRTELDLRPQGLELFENTSAPYQLQTFVLPATPPQLVSPRVLEVD TQGTVVCSLD<br>GLFPVSEAQVHLALGDQRLNPTVTYGNDSFSAKASVSVTAEDEGTQRLTCAVILGNQSQE<br>TLQTVTIYSFPAPNVILTKPEVSEGTEVTVKCEAHPRAKVTLNGVPAQPLGPRAQLLLKA<br>TPEDNGRSFSCSATLEVAGQLIHKNQTRELRVLYGPRLDERDCPGNWTWPENSQQTPMCQ<br>AWGNPLPELKCLKDGTFPLPIGESVTVTRDLEGTYLCRARSTQGEVTRKVTVNVLSPRY H<br>HHHHH
CD45	MTMYLWLKLLAFGFAFLDTEVFVTGQSPTPSPTGLTTAKMPSVPLSSDPLPTHTTAFSPA<br>STFERENDFSETTTSLSPDNTSTQVSPDSLDNASAFNTTGVSSVQTPHLPTHADSQTPSA<br>GTDTQTFSGSAANAKLNPTPGSNAISDAYLNASETTTLSPSGSAVISTTTIATTPSKPTC<br>DEKYANITVDYLYNKETKLFTAKLNVNENVECGNNTCTNNEVHNLTECKNASVSISHNSC<br>TAPDKTLILDVPPGVEKFQLHDCTQVEKADTTICLKWKNIETFTCDTQNITYRFQCGNMI<br>FDNKEIKLENLEPEHEYKCDSEILYNNHKFTNASKIIKTDFGSPGEPQIIFCRSEAAHQG<br>VITWNPPQRSFHNFTLCYIKETEKDCLNLDKNLIKYDLQNLKPYTKYVLSLHAYIIAKVQ<br>RNGSAAMCHFTTKSAPPSQVWNMTVSMTSDNSMHVKCRPPRDRNGPHERYHLEVEAGNTL<br>VRNESHKNCDFRVKDLQYSTDYTFKAYFHNGDYPGEPFILHHSTSYNSK HHHHHH
CD34 prec.	MLVRRGARAGPRMPRGWTALCLLSLLPSGFMSLDNNGTATPELPTQGTFSNVSTNVSYQE<br>TTTPSTLGSTSLHPVSQHGNEATTNITETTVKFTSTSVITSVYGNTNSSVQSQTSVISTV<br>FTTPANVSTPETTLKPSLSPGNVSDLSTTSTSLATSPTKPYTSSSPILSDIKAEIKCSGI<br>REVKLTQGICLEQNKTSSCAEFKKDRGEGLARVLCGEEQADADAGAQVCSLLLAQSEVRP<br>QCLLLVLANRTEISSKLQLMKKHQSDLKKLGILDFTEQDVASHQSYSQKT HHHHHH
PODXL	SPSPSPSPSPSQNATQTTTDSSNKTAPTPASSVTIMAT DTAQQSTVPTSKANEILASVKA<br>TTLGVSSDSPGTTTLAQQVSGPVNTTVARGGGSGNPTT TIESPKSTKSADTTTVATSTAT<br>AKPNTTSSQNGAEDTTNSGGKSSHSVTTDLTSTKAEHL TTPHPTSPLSPRQPTLTHPVAT<br>PTSSGHDHLMKISSSSSTVAIPGYTFTSPGMTTTLPSS VISQRTQQTSSQMPASSTAPSS<br>QETVQPTSPATALRTPTLPETMSSSPTAASTTHRYPKT PSPTVAHESNWAKCEDLETQTQ<br>SEKQLVLNLTGNTLCAGGASDEKLISLICRAVKATFNP AQDKCGIRLASVPGSQTVVVKE<br>ITIHTKLPAKDVYERLKDKWDELKEAGVSDMKLGDQGP PEEAEDRHHHHHHHHHH
PODXL2	GSDEPGPEGLTSTSLLDLLLPTGLEPLD SEEPSETMGLGAGLGAPGSGFPSEENEESRIL<br>QPPQYFWEEEEELNDSSLDLGPTADYVF PDLTEKAGSIEDTSQAQELPNLPSPLPKMNLV<br>EPPWHMPPREEEEEEEEEEEREKEEVEK QEEEEEEELLPVNGSQEEAKPQVRDFSLTSSS<br>QTPGATKSRHEDSGDQASSGVEVESSMG PSLLLPSVTPTTVTPGDQDSTSQEAEATVLPA<br>AGLGVEFEAPQEASEEATAGAAGLSGQH EEVPALPSFPQTTAPSGAEHPDEDPLGSRTSA<br>SSPLAPGDMELTPSSATLGQEDLNQQLL EGQAAEAQSRIPWDSTQV ICKDWSNLAGKNYI<br>ILNMTENIDCEVFRQHRGPQLLALVEEV LPRHGSGHHGAWHISLSKPSEKEQHLLMTLVG<br>EQGVVPTQDVLSMLGDIRRSLEEIGIQN YSTTSSCQARASQVRSDYGT HHHHHHHHHH
